# Committing to oral care support for older people living in long-term residential care settings: A theoretically informed scoping review of enablers and barriers influencing care staff behaviours

**DOI:** 10.1016/j.ijnsa.2026.100658

**Published:** 2026-08-13

**Authors:** Karen Spilsbury, Julia Csikar, Reena Devi, Karen Vinall-Collier, Magda Jordao, Judy Wright, Carl Thompson, Alys Wyn Griffiths, Paul Wilson, Edna Feenan, Karen Winterburn, Sharon Earnshaw, Gail V.A. Douglas

**Affiliations:** a School of Healthcare, University of Leeds, Leeds, UK; b School of Dentistry, University of Leeds, Leeds, UK; c School of Humanities and Social Sciences, Leeds Beckett University, Leeds, UK; d Leeds Institute of Health Sciences, University of Leeds, Leeds, UK; e Sheffield Institute of Translational Neuroscience, University of Sheffield, Sheffield, UK; f Centre for Primary Care and Health Services Research, University of Manchester, Manchester, UK; g NICHE-Leeds Family and Friends, University of Leeds, UK; h Westward Care, Leeds, UK

**Keywords:** Care homes, Caregivers, COM-B, Long-term care, Older adults, Oral health, Oral hygiene, Personal care, Residential care, Scoping review, Theoretical domains framework (TDF), Workforce

## Abstract

**Background:**

Poor oral health among older people living in long-term residential care is associated with serious health consequences and reduced quality of life. Oral health commonly deteriorates in these settings and is often attributed to care staff workload pressures and limited oral health knowledge and skills. However, practice colleagues in our established UK care home and science partnership told us that a range of factors support and hinder them with oral care provision.

**Objective:**

To identify and synthesise the individual (micro), organisational (*meso*) and wider social and political (macro) factors that influence oral care provision for older people living in long-term residential care.

**Design and method:**

A scoping review was undertaken. Search strategies were developed by information specialists and study selection involved independent screening by two reviewers using predefined eligibility criteria. Data synthesis was informed by the Capability, Opportunity, Motivation and Behaviour (COM-B) model and the Theoretical Domains Framework (TDF). Reporting followed the Preferred Reporting Items for Systematic Reviews and Meta-Analyses Extension for Scoping Reviews (PRISMA-ScR) guidelines.

**Data sources:**

Systematic searches were conducted in February 2022 and updated in March 2026. Searches included CINAHL, Web of Science Core Collection, Embase, MEDLINE, APA PsycInfo, Google Scholar and relevant grey literature sources.

**Findings:**

Sixty-three studies (reported in 86 publications) were included. The most frequently reported influences on staff behaviours were in the COM-B ‘opportunity’ domain: environmental context and resources, and social influences. Staff knowledge and skills were important determinants of capability, while beliefs about consequences influenced motivation. Key enablers included training, oral health champions, supportive leadership, assessment and monitoring systems, and collaborative relationships between care staff, residents, relatives and dental professionals. Key barriers included staffing shortages, workload pressures, limited time, inadequate training, low organisational prioritisation of oral care, and restricted access to external dental services. Interpersonal relationships were central to effective oral care provision.

**Conclusions:**

Oral care provision is influenced by a complex interplay of factors at the individual, organisational and policy levels and cannot be attributed solely to care staff. While workforce pressures and limited training are important, staff knowledge, skills and behaviours must be understood within a broader organisational and system context. Interventions should extend beyond technical training to address emotional labour, relational dynamics and structural constraints. The COM-B and TDF frameworks provide a robust foundation for designing multi-component, context-sensitive interventions that reflect the realities of long-term care environments.

**Protocol registration:**

Open Science Framework https://doi.org/10.17605/OSF.IO/STRKW

**Tweetable abstract:**

‘Mouth minutes’ matter. Poor oral care in care homes affects older adults' health & quality of life. Our theory informed scoping review maps barriers/enablers for oral care provision in this setting. It’s about more than technical skill #OralHealth #CareHomes #NICHELeeds @IJNS

**What is already known**

• Poor oral health in older adults living in long-term residential care is associated with serious health consequences, such as cardiovascular disease, respiratory tract infections, poor control of diabetes, malnutrition, increased mortality, and reduced quality of life.
• Oral care support in these settings is often inadequate and frequently attributed to the pressures care staff experience -the lack of time they dedicate to oral care or their deficiencies in knowledge and skills.
• A significantly higher number of older people will retain some natural teeth and this necessitates improved access to dental care, alongside care staff supporting older people in maintaining oral health and preventing problems.
• There is limited synthesis of the individual (micro), organisational (meso) and wider social and political (macro)factors that influence care staff behaviours to support oral care.

Alt-text: Unlabelled box dummy alt text

**What this paper adds**

• This is the first review to synthesise individual (micro), organisational (meso) and wider social and political (macro)factors that influence oral care for older people living in long-term residential care, offering opportunities to address factors at the right level.
• The review identifies a complex interplay of individual, organisational and policy-level factors influencing oral care support in long-term residential care settings.
• It highlights that poor oral care cannot be solely attributed to care staff and their technical skill and competence, as is sometimes assumed.
• Utilising behavioural science, the COM-B model and TDF provides a robust foundation for designing multi-component, context

Alt-text: Unlabelled box dummy alt text

## 1 Background

Long-term residential care, also internationally termed care homes or nursing homes, provides accommodation and 24-hour personal care and/ or nursing support for people with significant physical, psychological, or cognitive health and care needs. It is estimated that millions of older adults worldwide live in long-term care settings, with demand increasing sharply in the oldest-old population (LaingBussion, 2016; Harris-Kojetin et al., 2019; Statista Research Department, 2024). Many of these older people are living with complex health conditions, cognitive impairments and polypharmacy (Gordon et al., 2014) often alongside poor oral health (Moore and Davies, 2016; Wong et al., 2019). Poor oral health in older adults is associated with serious health consequences, including cardiovascular disease, diabetes, rheumatoid arthritis, cognitive decline, respiratory tract infections, malnutrition, and increased mortality (Kotronia et al., 2021; D’Aiuto et al., 2025; Zhu et al., 2025). As more older adults retain their natural teeth, oral care needs are increasingly important and more complex (Hellyer, 2011).

In most long-term care settings, daily oral care is provided by frontline care staff -such as care assistants, support workers and nursing staff - as part of routine personal care. However, responsibilities are not universal and vary across countries and care systems depending on staffing models, regulatory frameworks and availability of dental professionals. Understanding how to promote care staff behaviours to commit to effective ‘mouth minutes’ is important to maintain the oral health of older care home residents. Maintaining oral health is essential for residents’ physical wellbeing and also for quality of life – supporting confidence, communication and social engagement (Porter et al., 2015; van de Rijt et al., 2020). Daily oral care is therefore an important aspect of personal care support and involves approaches informed by knowing the resident, inspecting their mouth, removing plaque, cleaning oral tissues, using fluoride products, and maintaining oral tissue moisture (Coker et al., 2013).

Despite its acknowledged importance, oral care in these settings is often described as inadequate (Care Quality Commission, 2019). Several factors are cited as contributing to poor oral health in this population, including reduced manual dexterity which limits a resident’s ability to perform daily oral hygiene tasks, cognitive impairment and staffing shortages that constrain the time available for personal care. This may be compounded by staff not having much (if any) oral health training (Campbell et al., 2020).

Practice colleagues in our established UK care and science partnership, called Nurturing Innovation in Care Home Excellence in Leeds (NICHE-Leeds) (Griffiths et al., 2022), raised questions (in 2000) about how they could better support residents maintain their oral health. Recognising this requires commitment by care staff, they also highlighted many factors that support or hinder them with day-to-day oral care provision. Through participatory research we created a learning culture to address this important area of care and to effect local oral care practice changes (Griffiths et al., 2021). An outcome of this was to secure funding for national research to develop theory and research-informed guidance for care homes to promote staff behaviours to improve oral health and care for residents (Spilsbury et al., 2021). This guidance will be of international relevance (Weening-Verbee et al. 2024).

We have completed an overview of reviews to identify, appraise and sythesise systematic reviews of interventions or strategies provided by care home staff to support residents with their oral health (Csikar et al., 2025). Here, we present a scoping review of evidence on the factors that influence long-term care staff’s ability to provide and support oral care for older residents. Understanding the enablers and barriers to oral care provision is critical for improving practice in these settings, and for guiding policy.

## 2 Review question

The question addressed by this scoping review was:

*What are the individual (micro), organisational (meso) and wider social and political (macro) enablers and barriers to oral care in care homes?*

## 3 Methods

We followed scoping review methodology to identify and appraise the volume, scope and quality of literature (Arksey and O’Malley, 2005; Levac, 2010; Peters et al., 2021). We have used the Preferred Reporting Items for Systematic Reviews and Meta-Analyses Extension for Scoping Reviews checklist (PRISMA-ScR) to guide reporting of our review (Tricco et al., 2018). The Capability, Opportunity, Motivation Behaviour model (COM-B) (Michie, van Stralen and West, 2011) and Theoretical Domains Framework (TDF) (Cane, O’Connor and Michie, 2012) were utilised to frame and interpret the evidence.

The review protocol was registered with the Open Science Framework prior to the review being conducted (https://osf.io/strkw/).

### 3.1 Eligibility criteria

Study eligibility was identified using the Joanna Briggs Institute (JBI) framework (Peters et al., 2021) (Table 1). We defined long-term residential care as a communal living setting providing long-term 24-hour personal care for people who need this support, including nursing care. Definitions of ‘older people’ vary across countries and contexts. To ensure inclusivity, we did not impose an age threshold: we included all studies that focused on populations that were identified by study authors as involving older people aged over 65 years and over.

**Table 1:** Eligibility criteria.

Domain	Inclusion criteria	Exclusion criteria
Participants	Older people (aged over 65 years) in long-term residential care settings	Younger populations; older people in short-term or acute care settings
Concept	Enablers/ barriers for care staff providing daily oral care: micro, *meso*, macro-level influences	Oral care solely delivered by dental professionals
Context	Residential and nursing homes	Short-term or acute care settings
Evidence sources	Primary studies	Opinion pieces editorials or reviews (without primary data)
Date range	Published from 2010 onwards	Published before 2010
Language	All languages	Not restricted by language

Enablers and barriers were defined as factors which support or prevent care staff carrying out and supporting older people with oral care (and health). This includes care staff practices which contribute to the prevention, arrest or slowing of dental diseases (such as routine toothbrushing), screening of residents’ oral health needs, or actions to support residents (such alleviating oral pain or mouth dryness).

All primary studies published in English from 2010 were included as: (i) this period followed a World Health Organization appeal for action on oral healthcare of older adults (Peterson et al., 2010); (ii) there has been a significant shift in the characteristics of residents and staff in these settings over the last decade (Centre for Policy Ageing, 2012), and evidence before 2010 was deemed less representative of the current population and context.

### 3.2 Search strategy

The search strategy was developed (JW) and peer reviewed (NK) - both information specialists - based on four key concepts: care homes, oral care, barriers and enablers, and qualitative studies. Qualitative sensitive search filters were used for OVID, MEDLINE and CINAHL (DeJean et al., 2016), adapted for use in EMBASE and PsycINFO and for Web of Science Core Collections (SSCI DeJean filter was adapted).

The searches were conducted in two phases: an initial search in 2022 and an update search in 2026. Five databases were searched (15 February 2022): CINAHL (EBSCOhost); Web of Science Core Collection (SCI-EXPANDED, SSCI, A&HCI, CPCI-S, CPCI-SSH, ESCI); Embase Classic and Embase (Ovid); MEDLINE (Ovid); and APA PsycInfo (Ovid). On 15 March 2022 we searched Google Scholar, screening the first 100 results. Unpublished (grey) literature was identified using simplified search strategies and manual browsing for relevant studies during the period 15 March to 4 April 2022. Sources included: British Society of Gerontology (https://www.britishgerontology.org/); Electronic Theses Online Service

(EThOS) (https://ethos.bl.uk/Home.do), and International Association for Dental Research Abstract Archives (IADR) (https://iadr.abstractarchives.com/search). The database searches were updated on 26 March 2026. Original search strategies and eligibility criteria were retained, and searches were limited to records added to the databases since the initial search date.

Searches were not restricted by language. Search results were deduplicated, and records uploaded to Rayyan (rayyan.ai) for screening.

### 3.3 Study screening and selection

Reviewers (MJ, JC, RD, KVC) independently pilot-screened the titles and abstracts of 50 records for eligibility. This pilot led to refinements in the criteria to improve precision and reduce ambiguity.

Following consensus on eligibility criteria, all remaining records were screened (title and abstract) and full papers selected from these by at least two reviewers (MJ, JC, RD, KVC, SE, KS, CT). For consistency, one reviewer screened and selected all records with another reviewer (MJ with JC, RD, KVC, SE or KS with CT). Discrepancies were resolved through discussion with a third team member. Full texts for included records were retrieved. For comprehensiveness, the reference lists of a sample of included studies were screened for any additional eligible studies: none were identified. Records identified through the updated search were screened using the same eligibility criteria and review procedures.

Although the search strategy did not restrict by language, only studies published in English were eligible. No translation of non-English papers was undertaken.

### 3.4 Data charting

Data charting was guided by JBI methodology (Peters et al., 2021). A data extraction table and codebook were developed and refined during a piloting phase: two reviewers independently extracted information from a sample of 10 included studies. Following the pilot, one reviewer (MJ or KS) completed data extraction for each study, identifying enablers and barriers. See Table 2, Table 3.

**Table 2:** Data extraction to describe included studies.

Data	Definition
Study ID	Generated for each study and to link study references
Reference	Reference(s) of study report(s)
Year	Year of publication.
Publication title(s)	Title of the records associated with the study.
Country	Country where the research took place
Study design	Select from the dropdown menu (based on NICE https://www.nice.org.uk/process/pmg4/chapter/appendix-d-glossary-of-study-designs and https://www.cebm.ox.ac.uk/resources/ebm-tools/study-designs): **Before-and-after:** Also called pre-post study design. The dependent variables are measured before and after an intervention has been delivered. **Case–control:** A comparative observational study in which the investigator selects people who have an outcome of interest (for example, developed a disease) and others who have not (controls), and then collects data to determine previous exposure to possible causes. **RCT**: Randomized controlled trial where participants are randomly allocated to receive either intervention or control. **cRCT:** Cluster randomized controlled trial where clusters are randomly allocated to receive either intervention or control. **Cohort:** An observational study in which a group or 'cohort' of people are observed over time in order to see who develops the outcome of interest **Cross-sectional:** A cross-sectional study is an observational study in which the source population is examined to see what proportion has the outcome of interest, or has been exposed to a risk factor of interest, or both. Cross-sectional studies are generally used to determine the population prevalence of outcomes or exposures. An approach that is often called a survey or a prevalence study. **NRCT:** Non-Randomized controlled trials are trials where participants are allocated to receive either intervention or control (or comparison intervention) but the allocation is not randomised **Case report:** A detailed report of the symptoms, signs, diagnosis, treatment, and follow-up of an individual patient **Case series:** A coherent and consecutive set of cases of a disease (or similar problem) **Feasibility study:** Feasibility Studies are pieces of research done before a main study in order to answer the question "Can this study be done?". They are used to estimate important parameters that are needed to design the main study (based on NIHR glossary, https://www.nihr.ac.uk/glossary?letter=F&postcategory=-1) . **Other:** if none of the above fit, enter your notes about this in the following column
Study aim/research question	Extract a quote containing the description of the study aim. If this is not available extract the description of the research question. If none are provided, insert ‘not available’.
Theoretical/conceptual framework	Record theoretical or conceptual framework clearly used in the study. This framework must be at the core of the study (and not just be a passing reference). If a particular theoretical or conceptual framework is not clearly stated write "Not clear"
Care context and type of care	Extract a quote describing of the context where the research is taking place (include type of care when described). Include any other relevant details.
Methods	Select from the drop-down menu (Based on NICE https://www.nice.org.uk/process/pmg4/chapter/appendix-d-glossary-of-study-designs): **Qualitative survey**: Qualitative surveys try to elicit qualitative data from surveys or questionnaires by providing open ended questions that try to encourage a lengthy response and then treat this data as qualitative data by coding and analysing it. **Quantitative survey/questionnaire**: Set of pre-defined questions and answers that allows to quantify how many people select certain answers/characteristics/opinions **Focus group**: A specific type of interview study where a group of people (usually 6–12) is interviewed by 1 or more facilitators or interviewers. The method explicitly includes and uses the group interaction to generate data. **Interview**: A qualitative method of data collection where participant's views are elicited via verbal interviews. Interviews can be structured (that is, reliant on set questions), semi-structured (the interviewer has a general idea of topics and themes to cover and will steer the interview towards these participants) or unstructured (the format and direction of the interview is set by the participant). **Non-participant observation**: A qualitative methodology where the researcher observes participants as they engage with the phenomenon being researched. Participant observation: A qualitative methodology where the researcher joins in with the participants as they engage with the phenomenon being researched. **Mixed-methods**: uses quantitative and qualitative methods in a single or multiphased study (Tashakkori & Teddlie, 1998) at all or many research stages (Creswell, 1995) including sampling strategies, data collection & analysis, findings synthesis, and integration & reporting. The data collected, analysed and synthesized can be numerical, but also textual / visual / multimedia data (based on https://www.rds-wm.nihr.ac.uk/mixed-methods-definitions). **Other**: if none of the above fit, enter your notes about this in the following column
Methods notes	Additional information about methods that does not fit in the previous definitions
Residents	Provide a brief description of the residents and sample size if available.
Care staff	Provide a brief description of the care staff participating in the research and sample size if available.
External staff	Provide a brief description of external (to the care home) practitioners participating in the research and sample size if available.

**Table 3:** Data extraction of enablers and barriers.

Category	Subcategory	Enablers references*	Barriers references*
**Micro level**	**Staff**		
	Staff knowledge about the resident	A22a-c; A29a-d; A48	A3; A22a-c
	Staff knowledge and awareness	A10; A11; A13; A15; A25; A32; A33; A34a-b; A38; A45a; A46; A49; A50; A51; A53; A54a; A54b; A55; A56a,b,c; A57; A58; A59; A60; A61; A62b; A63a,b	A11; A15; A24; A25; A26a-c; A29a-d; A31; A34a-b; A38; A40; A41; A45a; A45b; A46; A47; A48; A50; A52; A53; A54a; A54b; A56a,b,c; A58; A59; A60; A61; A62a,b; A63a,b
	Staff skills and experience	A5; A19; A28; A32; A44a-c; A46; A50; A51; A53; A54b; A56a,b; A58; A59; A60; A61	A1a-b; A11; A13; A14; A18; A26a-c; A29a-d; A30; A38; A46; A47; A48; A51; A52; A53; A54b; A56a,b; A59; A60; A61
	Staff communication techniques	A16a-b; A18; A22a-c; A41; A58	A19
	Promotion of residents’ independence	A8; A25	A40
	Staff personal responsibility	A15; A29a-d; A33; A46; A48	A15; A33; A48
	Staff self-efficacy / Confidence	A1a-b; A14; A32; A33; A44a-c; A46; A49; A50; A51; A53; A56a,c; A58; A59; A60; A61; A62b; A63b	A1a-b; A7; A26a-c; A46; A49; A51; A53; A56a,c; A59; A60; A61; A62b; A63b
	Staff attitude towards change		A6; A10; A62b
	Staff attitudes and beliefs about providing oral care to residents	A9; A11; A15; A18; A25; A40; A44a-c; A45a; A46; A50; A51; A53; A54a; A54b; A55; A56a,b,c; A58; A59; A60; A61; A63a,b	A3; A6; A7; A9; A10; A11; A14; A15; A16a-b; A25; A29a-d; A30; A31; A35; A40; A45b; A46; A47; A49; A52; A53; A54a; A54b; A56a,b,c; A59; A60; A61; A63a,b
	Staff attitudes and beliefs their own oral health	A14; A45a	A2; A27a-b; A45a
	Staff incentives/Sanctions	A10; A11; A31	A11
	Staff memory processes		A32
	Staff motivation	A10; A26a-c; A29a-d; A40; A45a; A49; A53; A56c	A3; A11; A13; A14; A45b; A53; A56c
	Staff emotion	A49; A53	A9; A18; A22a-c; A35; A40; A41; A49; A53; A55
	Staff self-monitoring		A18
	**Residents**		
	Resident admission status	A40	A10; A40; A63b
	Resident attitudes and beliefs	A29a-d; A62a	A10; A16a-b; A24; A29a-d; A38; A46; A60; A62a
	Resident autonomy/ independence	A46; A56b; A58; A62a	A40; A45a; A46; A47; A52; A53; A55; A56b; A62a A58
	Resident cognitive abilities		A10; A14; A21a-b; A25; A46; A47; A48; A52; A53; A54a; A54b; A56a,b; A57; A58; A59; A60; A61; A63b
	Resident financial status		A8
	Resident knowledge and awareness	A62a	A24; A26a-c; A62a
	Resident physical status		A18; A40; A45a; A46; A47; A51; A52; A54b; A55; A57; A59; A60; A61; A62a
	Resident sense of self	A16a-b	A16a-b; A17; A31
	Resident communication ability		A40; A45a; A46; A47; A48; A52; A53; A55
	Resident personal preferences and needs	A22a-c; A46; A61	A22a-c; A61
	Residents' trust in the carer		A24
	**Relatives**		
	Relatives provide oral care		A25
	Relatives’ attitudes and beliefs		A25
	Relatives’ knowledge and awareness	A26a-c; A31; A38; A62b	A26a-c; A31; A62b
	Relatives’ monitoring	A62a	A25; A62a
	Relatives’ support to access dental services	A17; A62a	A38; A62a
	Relatives’ support with oral care supplies		A8; A17; A18; A25
	**Relationships**		
	Staff-External HCP collaboration	A12; A18; A25; A26a-c; A29a-d; A46; A47; A50; A53; A54a,c; A57; A59; A60; A61; A62b; A63b	A1a-b; A24; A29a-d; A40; A46; A52; A53; A54a,b; A57; A59; A60; A61; A62b; A63b
	Staff-relatives collaboration	A8; A25; A50; A52; A53; A61	A26a-c; A52; A53; A55; A61
	Staff-residents collaboration	A1a-b; A2; A17; A26a-c; A29a-d; A34a-b; A50; A54a; A54b	A1a-b; A2; A8; A9; A10; A18; A19; A25 A21a-b; A26a-c; A30; A31; A34a-b; A38; A40; A41; A54a; A54b
	Staff-resident-relatives collaboration		A8; A17; A18; A29a-d
	**Team**		
	Peer to peer support	A10; A18; A22a-c; A46	A22a-c
	Roles and responsibility	A45b; A46; A47; A50; A53; A54a; A54b, A56b,c; A57; A58; A59; A61	A6; A29a-d; A38; A46; A47; A48; A50; A52; A54a; A54b; A56b,c; A57; A59; A61
	Team culture	A29a-d; A46; A50; A53; A54a; A54b; A56c; A57; A58; A59; A60; A61; A62b	A29a-d; A53; A54a; A54b; A56c; A57; A58; A59; A60; A61; A62b
**Meso level**	**Training**		
	Coaching		A5; A15; A18; A28; A39
	Training format	A1a-b; A10; A30; A31; A32; A50	A3; A30; A32
	Training frequency	A1a-b; A10; A38; A44a-c	A38; A45b
	Training provision	A1a-b; A2; A3; A4a-b; A5; A6; A7; A8; A9; A10; A13; A14; A20; A21a-b; A24; A26a-c A27a-b; A31; A33; A35; A38; A39; A40 A41; A43; A44a-c; A46; A47; A49; A50; A51; A53; A54a; A54b; A56a,b,c; A57; A58; A59; A60; A61; A62b; A63a,b	A2; A3; A9; A10; A21a-b; A26a-c; A30 A31; A35; A41; A45a; A45b; A46; A47; A48; A49; A50; A51; A52; A53; A54a; A54b; A55; A56a,b,c; A57; A58; A59; A60; A61; A62b; A63a,b
	Training resources	A22a-c; A30	A3; A13; A30
	Training time		A3; A30; A32
	**Management**		
	Management attitudes and beliefs on oral care	A26a-c; A30; A51	A2; A8; A26a-c; A30
	Management knowledge and awareness on oral care	A26a-c; A47; A51	A26a-c; A45b
	Management leadership	A6; A28; A29a-d; A30; A44a-c; A45b; A47; A50; A54a; A54b; A56a,b,c; A57; A58; A59; A60; A61; A62b; A63a,b	A1a-b; A6; A45b; A46; A47; A52; A54a; A54b; A56a,b,c; A57; A60; A61; A62b; A63a,b
	**Care organisation**		
	Diet and nutrition	A8; A56a-c	A14; A46; A54b; A56a,b
	Monitoring	A15; A18; A23; A28; A31; A46; A47; A50; A51; A54b; A58; A59; A60; A61; A62a,b; A63b	A6; A11; A25; A28; A40; A45b; A46; A48; A54b; A59; A60; A61; A62a,b; A63b
	Oral care policy	A8; A18; A25; A29a-d; A30; A31; A33; A41; A50; A54b; A59; A60; A61; A62a,b	A3; A25; A27a-b; A29a-d; A31; A35; A36a-b; A38; A40; A45b; A47; A48; A52; A54b; A59; A60; A61; A62a,b
	Oral care tools	A3; A5; A6; A8; A10; A11; A20; A22a-c; A23; A28; A30; A33; A36a-b; A40; A55; A56a,b,c; A58; A59; A60; A61; A62a,b; A63b	A1a-b; A18; A22a-c; A28; A29a-d; A38; A46; A47; A55; A56a,b; A58; A59; A60; A61; A62a,b; A63b
	Oral health champion	A11; A18; A28; A29a-d; A31; A33; A41; A44a-c; A47; A50; A57; A58; A59; A61; A62b	A41; A57; A61; A62b
	Physical resources for oral care	A4a-b; A18; A28; A33; A38; A40; A53; A54b; A55; A56a,b; A58	A3; A11; A14; A22a-c; A28; A29a-d; A40; A41; A45a; A45b; A53; A54b; A55; A56a,b; A58
	Processes of change	A11; A29a-d	A6; A44a-c
	Standards of care	A34a-b	A3; A34a-b
	**Staffing and organisational structure**		
	Organisation characteristics	A44a-c	A27a-b
	Staffing/Human resources		A2; A6; A10; A11; A18; A25; A26a-c; A28; A29a-d; A31; A32; A34a-b; A35; A45a; A45b; A46; A47; A48; A49; A51; A52; A53; A54a; A54b; A55; A56a; A57; A58; A59; A60; A61; A62a,b; A63a,b
	Time	A1a-b; A34a-b; A57	A1a-b; A9; A10; A11; A14; A17; A18; A21a-b; A22a-c; A25; A30; A31; A32; A34a-b; A40; A45a; A45b; A46; A47; A48; A49; A51; A52; A53; A54a; A54b; A55; A56a,c; A57; A58; A59; A60; A61; A62b; A63a,b
**Macro level**	**External Health Care Professionals**		
	Access to dental services	A3; A8; A24; A26a-c; A31; A34a-b; A38; A47; A50; A53; A54b; A56a,b; A57; A59; A60; A61; A62a,b; A63b	A3; A8; A14; A26a-c; A24; A31; A34a-b; A38; A40; A45b; A48; A52; A53; A54a; A54b; A56a,b; A57; A60; A61; A62a,b; A63b
	External HCP support in training and mentoring	A46; A47; A50	A1a-b; A6; A11; A12 A15; A29a-d; A37; A40; A45b; A46; A52
	External HCPs inter-professional collaboration		A11; A29a-d
	Oral care professionals providing oral care		A1a-b; A2; A18; A26a-c; A37
	Oral care professionals’ attitudes and beliefs		A26a-c
	Oral care professionals’ relationship skills	A24	A24
	**Policy**		
	Funding and costs	A11; A44a-c	A8; A26a-c; A31; A44a-c; A45a; A45b; A54a; A54b; A57; A61; A62a
	Higher education contribution to policy		A27a-b
	National/Regional monitoring		A3
	National/Regional oral care policy	A3; A10; A11; A22a-c; A27a-b; A57; A58	A22a-c; A29a-d; A45b; A57; A58
	Policy makers’ attitudes and beliefs		A29a-d
	Public awareness		A26a-c; A29a-d; A52
	Stakeholder involvement		A11; A29a-d; A30

*See Appendix 1 for references.

Due to the high volume of included studies identified from 2010 onwards, we agreed to prioritise studies published in the 5 years preceding the original search (February 2017 to February 2022). After completing data extraction for this subset, we reassessed the need to continue. We determined that the extracted data already covered a broad range of topics and contexts and that further extraction was unlikely to yield additional insights. Data charting was concluded at this point (44 studies; 61 reports). Studies identified through the updated search (February 2022 to March 2026) were charted using the same extraction framework and procedures. Following the updated search (2022–2026), the final review included 63 studies, represented in 86 reports.

Although quality assessment was initially included in our protocol, the piloting phase indicated that methodological quality did not affect our ability to identify reported enablers and barriers. Because our review aimed to map the breadth of influences on oral care, rather than assess effectiveness or make judgements about the strength of evidence, quality appraisal would not have meaningfully contributed to answering our research question. This approach aligns with established scoping review methodology, which notes that critical appraisal is optional and should be used only when directly relevant to the review’s purpose (Peters et al., 2021).

### 3.5 Synthesis and consultation

We followed an iterative approach to data analysis. Initially enablers and barriers were organised according to system levels – micro, *meso* and macro. We developed subcategories under these broad levels. At the micro level we categorised enablers and barriers related to care staff, residents, relatives or external staff. We identified a distinct group of enablers and barriers that centred on relationships between staff, residents and relatives, rather than individuals. At the meso‑level we grouped the enablers and barriers to four categories: organisational structure, management, care organisation and oral care training for staff. At the macro-level we grouped enablers and barriers to national or regional polices and external health care professionals.

**Fig. 1:**
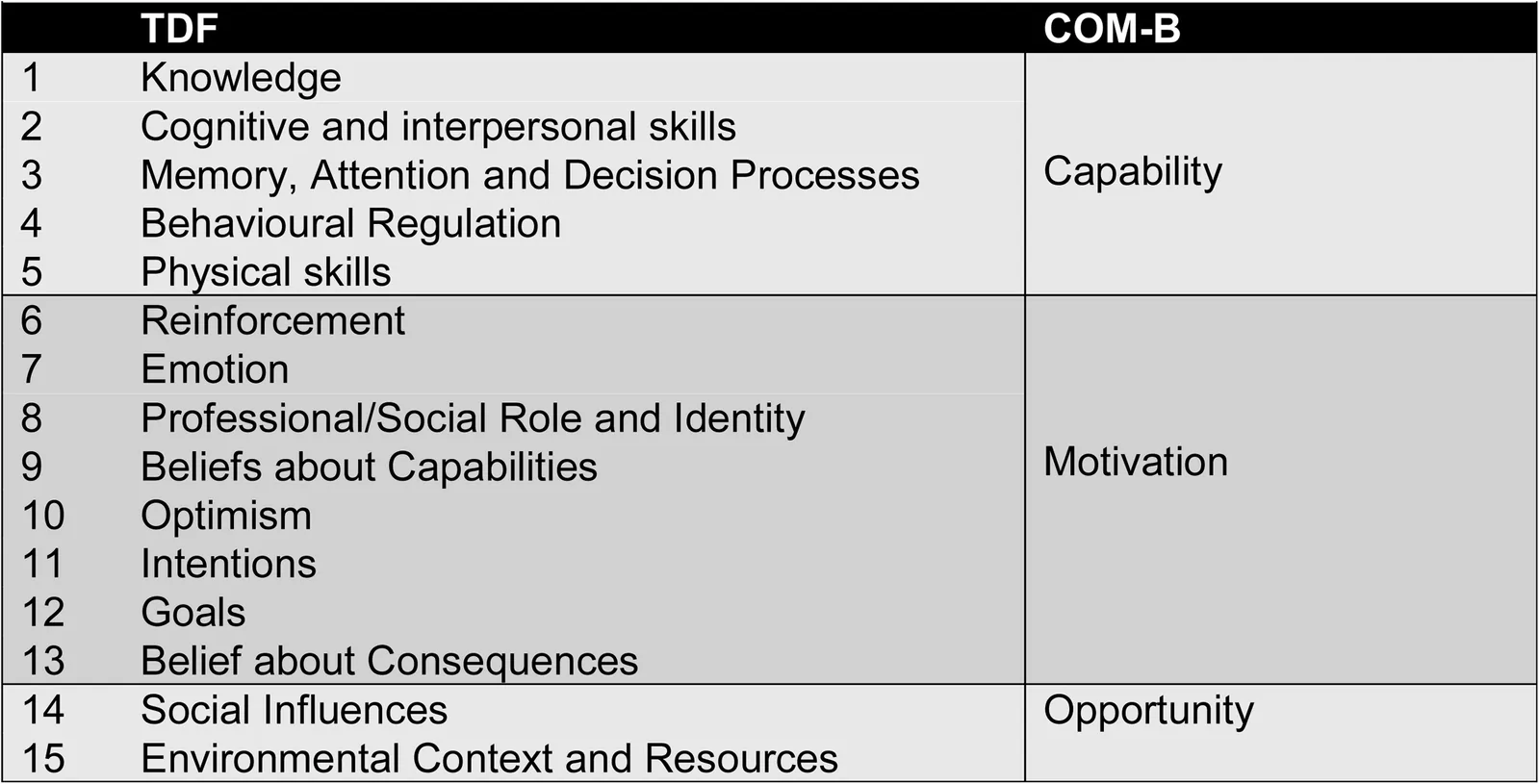
TDF + COM-B.

Our analysis was informed by the TDF (Cane, O’Connor and Michie, 2012) and its connection to the COM-B model (Michie, van Stralen and West, 2011). We used the 15 TDF domains to label categories of enablers and barriers, maintaining flexibility and induction to analyse these data. The TDF domains are listed in Fig. 1. These domains map to the COM-B model: capability (TDF domains 1 to 5); motivation (TDF domains 6 to 13; and opportunity (TDF domains 14 and 15).

The categories and subcategories were presented to 4 consultation groups comprising oral care and long-term care specialists, care staff, and residents and their relatives. Visual representations of our data were used to support these consultations (an example is provided in Fig. 3). Ethical approval for the consultations was granted by the School of Healthcare Research Ethics Committee, University of Leeds (HREC 21-004). The consultations were particularly valuable to refine the *meso* and macro level subcategories. Findings from these consultations are reported by Vinall-Collier et al. (2026).

A narrative synthesis was used to organise and present our scoping review findings, as well as investigating similarities and differences between included studies. This approach is consistent with scoping review methodology (Peters et al., 2021), aiming to reflect breadth of evidence rather than its interpretation. Our findings are presented under the three system levels – micro, *meso* and macro – and subcategories, with the TDF domains embedded in the narrative (see Fig. 1). So, for example, (***TDF1***) indicates that the reported result aligns with the knowledge domain, or (***TDF1,13***) aligns with (i) knowledge and (ii) beliefs about consequences.

## 4 Findings

**Fig. 2:**
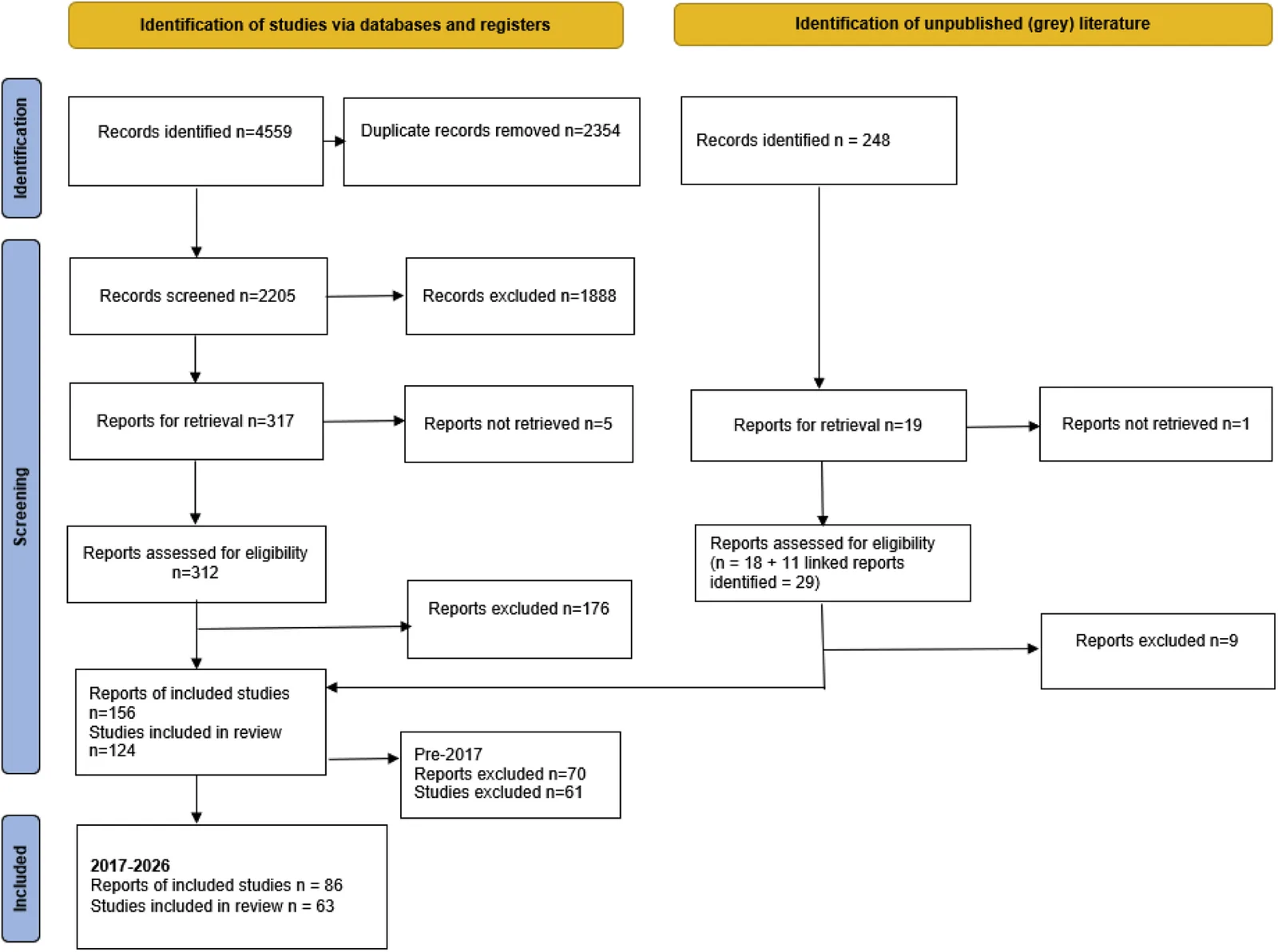
PRISMA diagram.

The complete screening and selection process is summarised below and presented in Fig. 2.

**Fig. 3:**
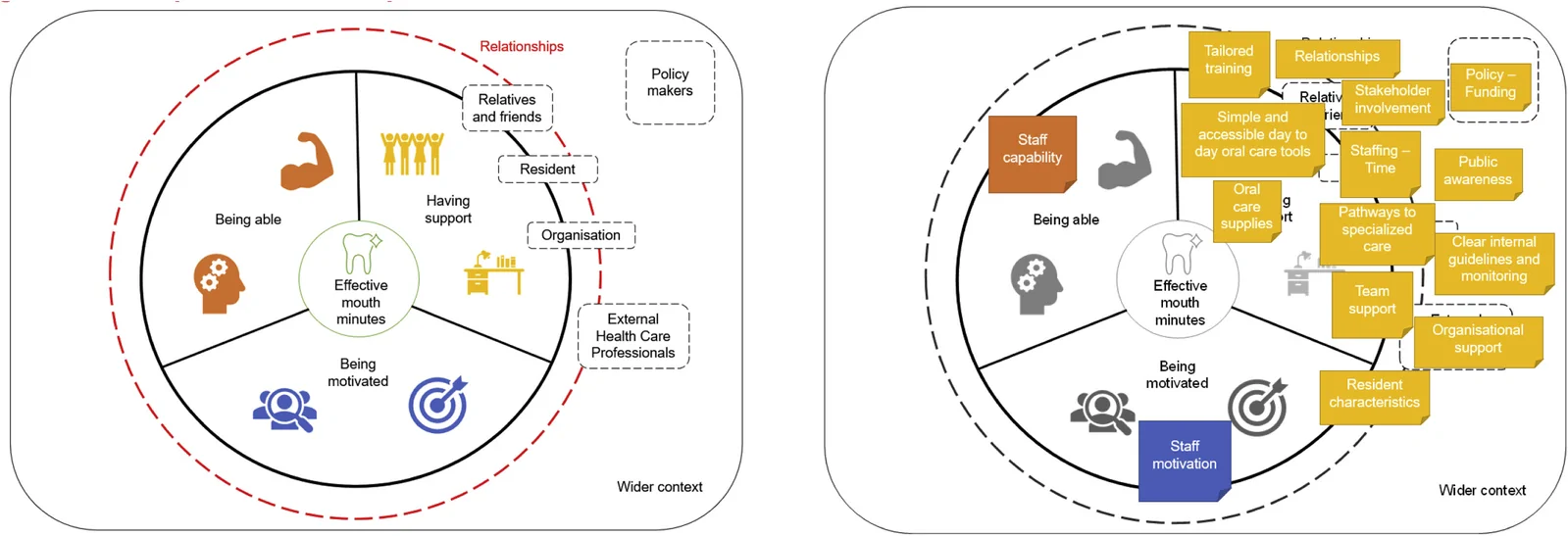
Examples of visual representation used in stakeholder consultations.

A total of 6853 records were identified through database searches and imported into EndNote X9 (Clarivate Analytics, PA, USA) for deduplication. An additional 100 records were identified through Google Scholar. After removing duplicates (n = 2354), 2205 records remained and were screened based on title and abstract. Of these, 317 records were considered potentially eligible and full text articles were sought for retrieval; 5 reports could not be retrieved. Following full text review, 136 reports were included. From the unpublished (grey) literature, an additional 248 records were browsed. Of these, 19 records were considered potentially eligible and full text reports were sought; 1 report could not be retrieved. During full text analysis of the 18 reports, 11 linked reports were identified. Following full text review of these 29 reports, 20 reports were included. The total number of included reports 2017 to 2026 was 156 reports, reporting on 124 studies. Due to volume of reports (and as described above - *Data charting*), we prioritised inclusion of studies published from 2017 to 2026 (63 studies; 81 reports).

### 4.1 Characteristics of included studies

Sixty-three studies were included (Table 4 and Appendix 1). The majority were conducted in high-income countries, the largest number from the US (n = 13), followed by the UK (n = 8) and Australia (n = 5). Other studies originated from Canada, and Sweden (each n = 4), Finland, Germany, Japan, China, Belgium and Malaysia (each n = 3), the Netherlands (n = 2), and a diverse range of other countries, including Brazil, Chile, Iceland, Iran, New Zealand, South Korea, Spain, South Africa and India (each n = 1). The type of long-term care setting varied: 21 studies focused specifically on nursing homes, while the remaining 42 included a mix of long-term care settings.

**Table 4:** Description of included studies.

Study ID	References	Country	Aim	Design	Framework	Methods	Sample: residents or relatives	Sample: staff	Sample: external health care professionals	Care setting
1	A1a, A1b	US	To assess the confidence of direct patient care professionals (DPCP) in completing section L (Oral/dental status) of file Minimum Data Set (MDS) and identified self-reported barriers. To examine the basis of the DPCPs’ oral health care knowledge in correctly identifying MDS oral health concerns. To explore [training in oral health] current adequacy.	Cross sectional	-	Quantitative survey	-	19 DPCP familiar with MDS reporting (7 excluded for lack of MDS experience)	-	26 long-term care facilities in Virginia
2	A2	Australia	To explore African migrant carers’ perceptions of oral health and their perspectives of oral care for aged care residents	Cross sectional	-	Interview	-	15 care staff	-	Residential aged care settings
3	A3	UK	To identify barriers to provision of oral healthcare for older people in care home settings in County Durham	Cross sectional	-	Mixed methods: quantitative survey and focus group/ interview	-	93/145 responses to survey by care home manager. 28 focus group/interview participants: care home manager, care staff; domiciliary care dentists; regional oral health improvement staff	Yes, but numbers in focus groups not clear	Residential and nursing homes
4	A4a, A4b	Germany	To investigate the long-term effectiveness of oral health education of caregivers in nursing homes with care-dependent and cognitively impaired residents	Cluster trial, not randomised	-	Measurement of clinical indicators of oral health	Residents at baseline: 178 intervention, 91 control. Residents at 6 months 156 intervention, 67 control. Residents at 12 months, 116 intervention, 40 control	Not clear how many staff received education in intervention group	-	14 nursing homes
5	A5	Australia	To explore the feasibility of integrating new multicomponent intervention protocols into nurse care plans; To enable nurses to select and schedule multicomponent preventive interventions throughout the day tailored to the individual as determined by nurse oral health assessment and testing	Feasibility study	-	Quantitative measures	8 residents	4 registered nurses	-	Nursing homes
6	A6	Sweden	To investigate first-line managers’ experience of and responses to a concise leadership intervention to facilitate the implementation of oral care clinical practice guidelines in nursing homes	Mixed methods	Medical Research Council framework	Interview and quantitative survey	-	5 managers and 8 registered nurses participated in interview. 96 registered nurses completed the survey	-	4 nursing homes
7	A7	Chile	To analyze oral health care related beliefs among caregivers of the institutionalized elderly in Antofagasta, Chile, 2019	Cross sectional	-	Quantitative survey	-	49 care staff	-	Long stay facilities
8	A8	New Zealand	To report New Zealand rest home managers’ opinions on access and barriers to oral health care for rest home residents.	Cross sectional	-	Interview	-	11 managers	-	Residential care
9	A9	Finland	To examine residential health care personnel’s opinions and attitudes toward older adults’ daily oral healthcare in long-term care facilities	Cross sectional	-	Quantitative survey	-	179 registered nurses, practical nurses and care staff	-	3 long term care facilities; 2 nursing homes and 1 residential home
10	A10	Australia	To examine aged care staff‘s views on the implementation of the Better Oral Health in Residential Care Training at their facilities, challenges that they faced in the provision of oral health care to residents and their training needs	Cross sectional	-	Interview	-	20 staff	-	13 long term care facilities
11	A11	UK	To describe how research and evidence influenced Welsh Government policy to fund a programme with the aim of improving the oral health of older people living in care homes; To describe how collaborative multi-agency working supported development and delivery of the programme.	Programme evaluation	-	Qualitative but not clear	-	-	-	287/650 care homes participating in training programme by year 4 of implementation
12	A12	Canada	To explore how the participation of dental hygiene students in interdisciplinary care conferences conducted in long-term care facilities influenced the staff’s awareness of oral health, the student’s provision of care as well as the student’s ability to work on an interdisciplinary team.	Cross sectional	-	Qualitative: focus groups and interviews	-	2 registered nurses; 1 director of care	6 health care professionals (physician, dietician, 2 recreational therapists, social worker, chaplain); 8 dental students	1 long term care facility
13	A13	Iran	To evaluate the effect of implementing the oral health care program on oral health status of elderly people resident in nursing homes	Before and after	-	Quantitative survey	101 residents (46 intervention group; 55 residents control group)	31 care staff	-	Nursing homes
14	A14	Finland	To analyse the implementation of oral health-related practices in private enhanced service housing units and nursing homes in Finland reported by supervisor nurses	Cross sectional	-	Quantitative and qualitative survey	-	245 registered nurses	-	Enhanced service housing and nursing homes
15	A15	Sweden	To examine the feasibility of an oral health coaching programme involving practical support on individual level to staff in a nursing home, aiming to improve oral health care‐related beliefs of nursing staff and the oral health of residents.	Feasibility study	Taxonomy of behavioural change methods	Quantitative measure and measurement of clinical indicators	46 residents	33 staff at baseline; 22 at follow-up	-	1 nursing home
16	A16a, A16b	UK	To explore residents' views and perspectives of dental care in care homes in order to understand how to deliver this care	Cross sectional	-	Qualitative interviews	26 residents	4 care home staff (including manager and care staff)	-	5 care homes
17	A17	UK	To explore care home residents’ views of dental care in care homes. The analysis focuses on the role of family and friends in supporting oral care.	Cross sectional	-	Qualitative interviews	26 residents	4 staff, including 1 care home manager, 1 team manager, 2 care staff	-	1 nursing home and 4 residential homes
18	A18	Canada	To assess oral care knowledge and challenges of nurses and allied nursing staff; To work with the nursing staff to identify and implement strategies that would help nursing staff provide optimal oral care for residents	Sequential mixed methods	Action Framework on Interprofessional Education and Collaborative Practice	Quantitative survey and focus groups	-	114 registered nurses and care staff completed the survey. 39 staff (including head nurses, registered nurses and care staff) participated in focus groups	-	1 large long term care facility
19	A19	South Korea	To examine whether the behavioural categories of care providers (person-centred and task-centred) were related to Resistiveness to Care; To examine which specific behaviours of care providers were related to Resistiveness to Care	Cross sectional	-	Quantitative measurment using non-participant observation of video recordings	23 residents	Unclear but provided care to sampled residents	-	4 long term care settings
20	A20	Germany	To develop a German version of the Oral Health Assessment Tool and to evaluate test–retest and inter-examiner reliability in use for the assessment of nursing home residents’ oral health by caregivers before and after dental training	Before and after	-	Quantitative	18 residents	4 care staff	-	1 nursing home
21	A21a, A21b	US	To assess and analyze the knowledge and attitudes of caregivers towards dental care for older adults in long-term care facilities; To train administrators, medical staff, and caregivers in the oral health competencies necessary to provide daily oral health care for Residents of Assisted Living Communities in Oregon.	Cross sectional	-	Quantitative survey	-	Study 1: 70 care staff. Study 2: 84 care staff pre-training survey and 132 care staff post training survey	-	Assisted living and aged care facilities
22	A22a, A22b, A22c	UK	To refine the complex oral health intervention (NICE guidance NG48) to ensure it is practically, clinically and culturally acceptable to care home staff and residents; To understand the context and mechanisms for delivery by exploring the challenges of providing oral care practices in care homes; To contribute to the embedment in best practice, thus translating the NG48 guideline into implementable practice.	Co-production	Double Diamond design process	Workshops	-	3 to 6 care staff per care home	-	3 care homes
23	A23	US	To investigate the effect of a checklist for oral care supplemented by random inspections by a charge nurse on improving oral hygiene	Mixed methods	-	Focus groups, intervention development and measurement of clinical indicators	32 residents (19 in intervention group; 13 in control group)	Unspecified number of staff in 3 focus groups (n = 6–8 per group)	-	Long term care facility
24	A24	Malaysia	To assess the perceptions of caregivers towards oral healthcare services received by elders in Malaysian nursing homes and to identify challenges and suggestions for improvement.	Cross sectional	-	Qualitative discussion using nominal group technique	-	21 care staff	-	Nursing homes
25	A25	US	To explore family member knowledge, beliefs, and expectations about oral health for assisted living residents with dementia, the role of family members in oral care in this setting, and their suggestions for future practice	Mixed methods	-	Quantitative survey and qualitative telephone interviews	23 family members: 16 children; 2 spouses; 2 siblings; and 3 other family member	-	-	9 assisted living facilities
26	A26a, A26b, A26c	US	To expand knowledge of the perceived benefits, barriers, and ability to perform or provide for oral health care and oral cancer screening as reported by Administrators and Directors of Nursing in Long-Term Care Nursing Facility; To propose empirically and conceptually supported interventions that might increase the capability and opportunity to provide of oral hygiene care and oral cancer screening in long-term nursing care facilities	Cross sectional	Health Belief Model and the COM-B model	Qualitative interview and focus groups	-	35 administrators or Directors of Nursing	-	Long term care nursing facilities
27	A27a, A27b	Spain	To measure the effectiveness of a 3-hour oral health training-programme provided to nursing-staff by assessing the residents’ oral health gains.	RCT	-	Quantitative survey; measurement of clinical indicators (baseline, 6 and 12 months)	369 residents	167 staff; 82 staff attended the training intervention	-	30 nursing homes
28	A28	US	To describe the design and preliminary evaluation of a Dementia-specific Oral Health Program, a multicomponent, interprofessional program developed to overcome multilevel challenges to existing direct care staff providing oral hygiene care in long term care settings	Mixed methods	Reach, Effectivenes, Adoption, Implementation, and Maintenance framework	Quantitative survey of staff; measurement of clinical indicators	11 residents	86 care staff – including registered nurses, licensed practical nurses and care staff - in total (not clear in which parts of the study)	-	Long term care unit
29	A29a, A29b, A29c. A29d	Netherlands	To synthesise a framework of barriers and facilitators in the normative integration of oral health care into general health care for frail older adults at macro (system), *meso* (organisation and interprofessional integration) and micro (clinical practice) levels	Cross sectional	Rainbow Model of Integrative Care	Interviews and focus groups	3 residents; 4 family members	4 registered nurses; 2 managers	5 dentists; 3 oral hygienists; 1 dental manager; 6 physicians	Nursing homes
30	A30	UK	To pilot co-production, delivery, and evaluation of an oral care training programme with care home staff	Co-production	Co-production (culture, structure, practice and review) as suggested by the Social Care Institute for Excellence	Quantitative and qualitative survey	-	87 care staff completed training but 45 completed the post training questionnaire	-	3 care homes
31	A31	Australia	To explore the perceptions of residential aged care nursing and management staff regarding oral care; To develop strategies to improve the oral health of aged care residents.	Cross sectional	-	Focus group	-	12 care staff: 5 managers and 7 registered nurses	-	2 residential care facilities
32	A32	Canada	To measure and understand the impact an online oral health education module has on the personal support worker provision of oral health care for residents in a long-term care facility	Before and after	Part of a multifaceted knowledge mobilisation intervention guided by implementation science	Mixed methods: questionnaire and focus groups/ interview	-	76 staff completed the questionnaire; 23 staff participated in focus groups and 4 staff interviewed	-	1 long term care facility
33	A33	US	To determine if an evidence-based oral care protocol in addition to a staff oral care training program on a LTC unit would increase staff knowledge of oral care in older adults and improve oral health outcomes in residents within a 14-day period. Other outcomes were to monitor compliance with documenting daily oral care two times per day for 14 consecutive days.	Before and after	Iowa Model of Evidence-Based Practice	Quantitative survey; measurement of clinical indicators; care documentation audit	10 residents	29 care staff, including registered nurses, licensed practical nurses and care staff	-	Long term care facility
34	A34a, A34b	Iceland	To assess oral care beliefs and oral hygiene procedures among nursing home personnel to identify strengths and weaknesses in managing oral care	Cross sectional	-	Quantitative survey	-	24 care staff, 64 practical nurses, 18 registered nurses	-	2 nursing homes operated by same organisation
35	A35	Brazil	To analyze the actions taken by caregivers of the elderly aimed at long-stay institutions for elderly residents in Brazil	Cross sectional	-	Interview	-	117 care staff	-	36 long stay institutions in 11 Brazilian municipalities
36	A36a, A36b	Japan	To develop and evaluate, with a dentist as gold standard, an oral health screening tool, the Oral Health Screening Tool for Nursing Personnel that assists long-term care facility nursing staff without preliminary training in identifying resident need for dentist referral.	Cross sectional	-	Quantitative measurement	-	57 residents 12 staff (4 registered nurses; 12 care staff)	-	A long-term care facility
37	A37	US	To explore the knowledge, attitudes, and practices of supervising nurse administrators regarding the oral care provided to long-term care facility residents and the role of dental professionals in those facilities.	Cross sectional	-	Quantitative survey	-	171 registered nurses	-	Skilled nursing facility
38	A38	Australia	To explore the perceptions of Residential Aged Care Facility (RACF) care staff towards the provision of oral health care following implementation of this new model of care. Specifically, this study sought to (i) examine the perceptions of care staff regarding oral health care practices in RACFs, and (ii) ascertain the needs and recommendations of care staff in relation to improving the delivery of oral health care in RACFs.	Cross sectional	-	Focus group	-	12 care staff	-	2 residential aged care facilities
39	A39	US	To discuss the implementation of a quality improvement project incorporating Mouth Care without a Battle, an evidence-based approach to person-centered daily mouth care, using a coaching model with part-time dental hygienists	Before and after	-	Quantitative measurement	440 residents	335 staff baseline; 227 follow-up (60% care staff, 22% licensed practical nurses; 12% registered nurses)	-	22 New York State nursing homes
40	A40	Netherlands	To explore attitudes, perceptions, and perceived barriers to and the perceived facilitators of daily oral health care and the actual daily oral health care performances among nursing home staff.	Cross sectional	-	Mixed methods: questionnaire and focus groups	-	409 registered nurses or nurse assistants. 14 focus groups but participant numbers unclear	-	21 nursing homes
41	A41	US	To improve direct care staff oral care knowledge, practices, beliefs, and attitude. To implement an evidence-based protocol. To assess protocol adherence. To examine staff barriers and facilitators to implementation at a single institution.	Before and after	-	Quantitative and qualitative survey	-	96 direct care staff participated, 70 completed post-test survey	-	Long term care facility
42	A42	Japan	To survey the oral health conditions of such older adults using the oral assessment sheet, developed in the current study for use by care workers for improving oral health conditions of older adults in care institutions To examine the assessment’s reliability and validity using multi-institution trials and the test-retest method before and after dental professionals’ guidance.	Cross sectional followed by before and after study	-	Quantitative survey	Survey 1: 188 residents. Survey 2: 45 residents	Survey 1: 79 care staff. Survey 2: 45 care staff	-	Survey 1: 13 nursing homes. Survey 2: 10 nursing homes.
43	A43	China	To determine whether improvement in caregivers‘ self-care oral hygiene skills would help them deliver better oral hygiene assistance to patients with Alzheimer’s disease at nursing homes and what components of training were critical for achieving favourable outcomes.	RCT	-	Quantitative survey, measurement of clinical indicators	146 residents	146 care staff		32 nursing homes
44	A44a, A44b, A44c	US	To evaluate the effectiveness of Mouth Care Without a Battle, a program that increases staff knowledge and attitudes regarding oral hygiene, changes mouth care, and improves oral hygiene, in reducing the incidence of pneumonia among nursing home residents	RCT	-	Measurement of clinical indicators	1108 residents in intervention group, 827 in control group	290 care staff in the intervention group and 144 in control group	-	14 nursing homes
45	A45a, A45b	South Africa	To explore oral health-related knowledge, attitudes and practices of caregivers and to establish coordinator perspectives on oral health provision in long-term care facilities.	Cross sectional and qualitative	-	Quantitative survey and qualitative interviews	-	188 caregivers;14 coordinators (4 managers and 10 nurses).	-	7 long-term care facilities
46	A66	Sweden	To describe healthcare workers' experiences of assessing oral health	Qualitative	-	Focus group interviews	-	28 nursing home staff (14 registered nurses and 14 nurse assistants	-	6 nursing homes for older people, including residents with dementia and extensive care needs
47	A47	Sweden	To investigate the views and experiences of professionals in leadership roles regarding oral health needs, routines, barriers and facilitators to oral care (linked with Senior Alert (SA) preventive care process and ROAG-J (Revised Oral Assessment Guide-Jönköping) oral health assessment framework)	Cross sectional	-	Web-based survey	-	464 staff in leadership roles (166 managers, 55 coordinators, and 243 registered nurses)	-	360 nursing homes (public and private)
48	A48	Canada	To explore the social organisation of oral care that contributes to poor oral health among residents in long-term care (LTC) homes.	Qualitative study	Institutional ethnography	Observations and interviews	-	Healthcare aides (96 h observation; 21 interviews); interviews with 3 RNs, 3 LPNs; 3 directors of care	-	2 long term care homes older people
49	A49	China	To investigate nurses' attitudes toward providing oral care in geriatric care facilities and identify associated factors, particularly work pressure, resilience and self-efficacy	Cross sectional	Psychological	Online survey	-	159 nurses	-	2 geriatric care facilities caring for older adults with varying degrees of dependence and cognitive impairment
50	A50	US	To describe and evaluate the MOTIVATE (Maine's Oral Team-Based Initiative Vital Access to Education)	Programme evaluation using a timed-series pre-post design	-	Three-wave evaluation: baseline (T1), post-module (T2), and post-implementation/follow-up (T3). Data collected through self-report surveys and qualitative (free-text) responses were analysed.	-	407 staff including certified nursing assistants, registered nurses, housekeeping staff, certified residential medication aides, administrators and other (dietary, maintenance and therapy staff)	-	6 Veterans’ long-term care facilities in Maine
51	A51	US	To assess caregivers' knowledge, attitudes and confidence regarding provision of oral hygiene assistance to nursing-home residents. Secondary objective: to assess administrators' knowledge and attitudes toward oral care provision and their confidence in caregivers' ability to provide oral care	Qualitative	-	Semi-structured face-to-face interviews	-	19 staff participants: 7 caregivers and 12 administrative staff	-	10 nursing homes in San Antonio, Texas
52	A52	Malaysia	To explore barriers to providing oral healthcare for older adults living in private residential aged care facilities	Qualitative	-	Qualitative interviews	-	Supervisors (n = 7)	-	7 private residential aged care facilities
53	A53	Germany	To assess possible health policy interventions for improving oral healthcare and oral hygiene in care homes by identifying barriers and facilitators using a behavioural change framework	Qualitative	Theoretical Domains Framework (TDF), COM-B (Capabilities, Opportunities, Motivations influencing Behaviour) model, and Behaviour Change Wheel (BCW).	Semi-structured interviews	-	8 care-home staff: 2 care-home managers, 4 section managers, 2 carers/nurses	3 dentists	2 rural care homes
54	A54a, A54b	UK	To evaluate the implementation and potential of role substitution (Dental Therapists and Dental Nurses delivering oral healthcare traditionally provided by dentists) to improve oral health among care-home residents	Process evaluation alongside the SENIOR cluster randomised controlled trial	PARIHS (Promoting Action on Research Implementation in Health Services) implementation framework	Semi-structured interviews	-	5 care-home managers.	16 healthcare professionals including Consultants in Dental Public Health, Consultants in Special Care Dentistry, dental professionals involved in preventive programmes in care homes and other relevant academics	43 care homes across England, Wales and Northern Ireland participating in the SENIOR trial (22 intervention, 21 control homes)
55	A55	Japan	To clarify how the COVID-19 pandemic changed the environment of nursing-home residents and the working environment, infection-control practices, and oral-health-care procedures of staff, and to explore regional differences	Cross sectional	-	Questionnaire distributed to staff in long-term care facilities in urban, suburban and rural regions	-	929 nursing-home staff: including 618 nursing care workers, 134 nurses, 38 care manager, and other staff n = 139)	-	40 long-term nursing care facilities
56	A56a, A56b, A56c	Finland	To investigate the implementation of oral-health practices, daily oral care, barriers/facilitators (antecedents), knowledge, attitudes, motivation and organisational factors affecting oral healthcare in nursing facilities from the perspective of supervisor nurses	Mixed programme: cross sectional and qualitative	-	Web-based questionnaire of supervisor nurses followed by demi-structured interviews with a sample	-	Supervisor nurses only: survey respondents n = 245; interview participants n = 19	-	Private enhanced service housing units and nursing homes across Finland
57	A57	Belgium	To explore care-home managers' perspectives on domiciliary dental care and identify barriers and facilitators to accessing professional oral healthcare for care-dependent residents	Qualitative	-	Semi-striuctured interviews	-	10 care home managers	-	6 care homes
58	A58	UK	To explore challenges of providing daily oral care in care homes, understand oral-care practices used by care staff, and co-design practical resources to implement NICE guideline NG48 for oral health in care homes	Qualitative co-design process conducted as part of the TOPIC (Improving the Oral Health of Older People in Care Homes) feasibility programme	Informed by Mindlines theory and the Consolidated Framework for Implementation Research (CFIR)	Co-design workshops	-	3–6 care-home staff per site from 3 care homes and 3 workshops	-	3 care homes from Sheffield, England
59	A59	Belgium	To explore how managers and caregivers perceive residents' oral health, identify oral-care needs, barriers to oral-care delivery, and current oral-health practices in long-term care settings	Cross sesctional	-	Two validated questionnaires (one for managers, one for caregivers)	-	342 staff participants: 145 managers and 197 caregivers (including nurses and nurse aides)	-	Long-term care organisations for older people, including residential and home-care services across Flanders, Belgium.
60	A60	Malayasia	To assess the oral health status of older adults living in long-term care and explore caregiver-reported challenges and recommendations for providing oral care	Mixed methods	-	Interviewer-assisted questionnaires, clinical oral examinations, plus focus group discussions with caregivers	115 residents aged over 60 years	16 caregivers	-	1 public long-term care facility
61	A61	India	To explore care managers' perceptions of the oral healthcare experiences, oral-health needs, barriers, facilitators, and service requirements of older adults living in residential aged care facilities	Qualitative	-	Online semi-structured interviews	-	10 care home managers	-	Residential aged care facilities (RACFs) in Western India
62	A62a, A62b	Belgium	To evaluate implementation and use of the Oral Health Section for interRAI (OHS-interRAI) in nursing homes, exploring resident perspectives on oral health and oral-care assessment, and caregiver experiences with implementation of routine oral-health assessment	Mixed methods	COM-B	Oral-health assessments using OHS-interRAI plus dental indices; in-depth resident interviews; focus groups, observations and implementation evaluation with non-dental caregivers during and after OHS-interRAI implementation	22 residents	31 staff interviewed; 19 caregivers observed completing 51 oral health assessments	-	3 nursing homes/ long-term care facilities
63	A63a, A63b	China	To evaluate the effectiveness and longer-term sustainability of an Oral Health Education Programme designed to staff knowledge, attitudes and practice for older residents in long-term care institutions	Intervention study with case control design	-	Intervention (oral-health education, oral-care skills training and return demonstration); questionnaires administered pre-intervention, post-intervention, and at 3- and 6-month follow-up. Oral-care delivery was additionally assessed through observation of staff providing oral care to residents	-	40 staff (20 intervention and 20 control)	-	Two long-term care institutions

Of the 63 included studies, over a third (n = 26) used a cross-sectional design, including surveys, interviews and focus group studies. Other study designs included mixed methods (n = 10), before and after/intervention studies (n = 8), randomised controlled trials (n = 3), feasibility studies (n = 2), co-production (n = 2), programme evaluations (n = 2) and one cluster non-randomised trial. Only 15 studies (24%) explicitly reported using a framework to guide their design or analysis, indicating a gap in theory-informed research in this area.

Participant groups studied included residents and their relatives, long-term care staff at different levels of seniority, external health and social care professionals. Staff were most frequently represented, in 57 of the 63 studies. In contrast residents were included in 18 studies, with relatives (5 studies) and external health and social care professionals (6 studies) less frequently included. Several studies (13 studies) included more than one participant group.

### 4.2 Understanding barriers and enablers for oral care

Barriers and enablers need to be understood as being operationalised at different levels - the individual and relationships (micro), the care home or care organisation (*meso*) and wider external influences (macro-level). Our findings are structured to present barriers and enablers at these different levels (summarised in Table 5). The studies were also mapped to the COM-B and TDF constructs (Table 6, Table 7). This mapping revealed a disproportionate focus on certain TDF constructs. Most frequent studied constructs were environmental context and resources, and social influences (opportunity). Knowledge and physical skills (capability) and beliefs about consequences (motivation) also feature prominently. Notably, optimism and goals – part of the opportunity domain - are absent from all studies. Other less studied barrier domains include cognitive and interpersonal skills; memory, attention and decision processes; behavioural regulation; reinforcement.

**Table 5:** Summary of enablers and barriers to oral care at micro, *meso* and maro-levels.

Level	Enabler and/ or barrier
**Micro (individual) level**	
***Staff***	Attitudes and beliefs
	Perceived role responsibility
	Oral care knowledge and skill
	Committed to promoting resident independence
	Cultural values and perceptions
	Communication skills
	Personal response to providing oral care
***Residents***	Oral health status
	Physical health status
	Mental and emotional well-being
	Cognitive abilities
	Attitudes and beliefs
	Communication skills
***Relatives***	Attitudes and beliefs
	Perceived responsibility to support family member and staff
	Oral care knowledge and awareness
	Willingness to provide resources and support access to dental care and services
	Communication skills
***Relationships***	Staff - resident
	Staff - relative
	Staff - staff
	Staff - external health care professionals
**Meso (care home) level**	
***Organisational structure***	Funding structure
	Staffing stability
***Care home management***	Attitudes and beliefs
	Prioritisation of oral care
	Involving staff to support changes in practice
***Care organisation***	Systems to monitor oral care provision
	Analysis of routine care data for monitoring and practice improvement
	Care home oral care policies and protocols
	Oral care plans
	Availability and accessibility of oral care equipment
	Physical space to support oral care
	Linking oral care with diet and nutrition provided for residents
***Oral care training for staff***	Dedicated time for oral care training
	Consideration of content, delivery, format, frequency and accessibility of training for staff
	Resources to support training and development
**Macro (policy) level**	
***National or regional policy***	Relevant and accessible oral care policies that are ‘fit for purpose’ for use by care home staff
	Monitoring oral care provision
	Understanding of care home context and population by policy makers
	Commissioning of dental care and services
***External professionals***	Access to dental care and services
	Attitudes and beliefs
	Communication skills
	Supporting student placements to share attitudes and beliefs of future professionals

**Table 6:** Enablers identified in studies mapped to TDF constructs.

COM-B domain	TDF constructs (number of studies)	TDF constructs not studied
**CAPABILITY**	Knowledge (24 studies) Physical skill (9 studies) Behavioural Regulation (9 studies) Memory, Attention and Decision Processes (6 studies) Cognitive and interpersonal skills (7 studies)	
**MOTIVATION**	Reinforcement (8 studies) Social/ Professional Role and Identity (16 studies) Beliefs about Capabilities (13 studies) Intentions (10 studies) Beliefs about Consequences (15 studies) Goals (7 studies) Emotion (4 studies)	Optimism
**OPPORTUNITY**	Social influences (28 studies) Environmental Context and Resources (45 studies)

**Table 7:** Barriers identified in studies mapped to TDF constructs.

COM-B domain	TDF constructs studied	TDF constructs not studied
**CAPABILITY**	Knowledge (26 studies) Physical skill (9 studies) Cognitive and interpersonal skills (11 studies) Memory, Attention and Decision Processes (5 studies) Behavioural Regulation (8 studies)	
**MOTIVATION**	Reinforcement (5 studies) Emotion (12 studies) Social/ Professional Role and Identity (10 studies) Beliefs about Capabilities (11 studies) Intentions (4 studies) Beliefs about Consequences (22 studies)	Optimism Goals
**OPPORTUNITY**	Social influences (29 studies) Environmental Context and Resources (55 studies)	

A narrative summary of our findings at the micro, *meso* and macro level, aligned with TDF domains, follows.

### 4.3 Micro (individual) level

#### 4.3.1 Staff

Oral care knowledge is crucial for staff capabilities to support residents (A10; A13; A15; A32; A33; A34a-b; A38; A45a; A46; A50; A51; A53; A56c; A58; A60; A61; A62b; A63a-b), as an enabler (when present) and barrier (when absent) (***TDF1,3***). Studies described the varied knowledge and understanding staff need to support residents with oral care. Staff understanding and familiarity with the care needs of individual residents was crucial (A22a-c; A29a-d; A46; A48), including knowledge of an individual’s medical conditions and medicines (A3). Promoting residents’ independence to care for their mouths for as long as possible was important (A8; A25) (***TDF13***). However, one study highlighted the negative consequences of promoting independence: residents sometimes choose not to care for their own mouths (A40) (***TDF13***).

Missing knowledge on specific areas, such as how to use oral care products (A41; A56a-b) or to assess a person’s mouth to screen for oral cancer (A26a-c) was highlighted as a barrier (***TDF1***). Knowing what oral care products to recommend for an individual resident, and when to refer to an appropriate health care professional, were important (A11; A50; A51; A56a) (***TDF1,8***). Gaining knowledge about oral health and care through training and education was the main enabler across multiple studies (A10; A13; A15; A25; A32; A33; A34a-b; A38; A45a; A46; A47; A50; A51; A53; A56a-c; A58; A63a-b) (***TDF1,15***): training and education is further considered under meso‑level.

Skills were most often reported to be acquired through practical experience; time ‘on the job’ offered the opportunity to develop capabilities in oral care provision (***TDF5,15***). Generally, more skills and experience were associated with better support for oral care, particularly for individuals with dementia (A28; A46; A58) and for undertaking oral health assessments (A1a-b; A5; A46; A50). However, somewhat contradictory, associations were found between less experienced staff and more positive attitudes toward oral care (A44a-c) (***TDF7,9***). Alongside physical skill, staff communication skills were considered a key enabler. Studies revealed the importance of staff attending to the comfort and needs of the resident (A19; A46), and several strategies could enhance communication, including negotiation (A16a,b), and distraction (A22a-c; A58) (***TDF2,15***). Acquiring communication skills and tips to encourage and support residents who demonstrate resistance to oral care was important for staff (A22c; A51; A58; A59). However, using strategies to control the care situation, for example standardising care routines rather than addressing individual needs or staff rationalising that poor oral health is inevitable in old age, could be a barrier to effective oral care for residents who demonstrate resistance (A19; A46; A58) (***TDF13,15***).

Staff attitudes and beliefs toward oral health and care (***TDF13***) significantly influenced their behaviour. Staff who practice good oral self-care and perceived their own oral health as important were more likely to provide better care for residents (A14; A45a). Conversely, staff who seek oral care only in an emergency tended to provide poorer oral care for residents (A2; A27a,b; A45a).

When staff felt personally responsible (and accountable) this supported oral care (***TDF8***); but a lack of personal responsibility led to neglect (A15; A33; A48). This responsibility was a strong motivational factor for staff to take the initiative with supporting oral care (A29a-d; A45a; A56). Related, negative attitudes and beliefs acted as barriers for oral care provision, including staff belief that: oral care is onerous (A9; A11; A47) (***TDF13***); difficult (A10; A14; A35; A47; A59) (***TDF9***); unpleasant (A3; A46; A53) (***TDF7***); less important than other aspects of personal care (A6; A25; A30; A40; A45b; A53) (***TDF 13***); and a deeply personal and intimate aspect of care (A16a,b; A46; A47) (***TDF14***). Staff emotions, including fear of hurting residents (A35; A41; A53) or being hurt or injured themselves (A18; A22a-c; A53), and disgust (A9; A18; A40; A46) were also barriers (***TDF7***). Cultural differences in staff attitudes towards oral care were identified (A30) (***TDF14***). Recognition and support from employers incentivised staff (A10; A11; A31; A50; A54b; A56c) (***TDF 6***). However, if staff believed their performance would be monitored, then staff resisted any training to avoid taking on such roles in a bid to minimise scrutiny (A11) (***TDF13,15***).

#### 4.3.2 Residents

The oral health status of a person when they move into the care home determines the level of support that staff needed to offer (A10; A40; A51; A62a-b) (***TDF13,15***). Oral care will be more difficult if oral health is already poor (A22c; A46; A47; A57). An individual’s oral health may be associated with their socio-economic status and ability to pay for care, both prior to and after moving into care (A8; A45b; A57).

A resident’s ability to communicate is key: when able to ask for help, care is more likely to be provided (A40; A48; A53) (***TDF2***). Personal preferences and needs, which can change rapidly for an individual, were identified as both an enabler and barrier for oral care provision (A22a-c; A46; A58). However, not all residents, due to cognitive abilities, recognise or express oral health and care as a priority (A34a; A46; A47; A48; A51; A52; A53; A56b; A58; A59) (***TDF2,13***). This translates into challenges due to the resident not understanding the need for daily oral care, and in other situations, refusing to wear or losing their dentures (A10; A14; A21a-b; A25; A46; A47; A52; A53; A56b; A58; A59) (***TDF1,7***).

The physical health status of a resident, including any comorbidities, may make the physical tasks associated with giving/receiving oral care more difficult because of pain or because the condition(s) take precedence over oral care activities (A18; A40; A45a; A47; A52; A57; A59) (***TDF5,13,15***). Further, the resident’s mental and emotional well-being is linked to oral health and willingness to engage with oral care (A16a-b; A17; A31; A55) (***TDF7,13***). Of particular importance, is the relationship between the resident and individual care staff (***TDF14***) (see below: Relationships).

Residents’ attitudes and beliefs (i.e. whether they consider oral health a priority) influenced their oral care behaviours (***TDF13***). When studies addressed residents’ attitudes and beliefs, this was most often in the context of barriers, such as not perceiving oral health as important, or considering this a personal and intimate topic that they did not want to openly discuss with staff (A16a-b; A46; A53) (***TDF7,13,14***). Conversely, interest and ownership of oral health by the resident led to consistent oral care and helped increase awareness among staff (A29a-d; A46) (***TDF14***). A resident’s level of oral health knowledge and awareness also exerts influence and can be both a barrier and enabler. For example, the individual may not see oral assessment as important (A26a-c; A46) (***TDF1,13***). or in contrast, seeks oral health education and assessment (A24; A46; A62a) (***TDF1***).

#### 4.3.3 Relatives

Relatives’ attitudes and beliefs towards oral health are key enablers for oral care (***TDF13,14***). Relatives recognising oral health as central to their family member’s well-being also recognised staff roles, and their own, when staff were ‘too busy’, to support oral care (A25; A52) (***TDF8***). A relative’s knowledge and awareness were positively associated with staff support for oral care; the relative was an advocate who monitored and alerted staff to oral care needs for their family member (A25; A50) (***TDF1,4,14***).

Where relatives were involved in making decisions about access to, and payment for, dental services, this impacted the care provided for the resident. In some circumstances, relatives booked a dental appointment and transport (A17; A57) (***TDF4,15***), while some opposed staff making arrangements for the resident to access dental services (A38) (***TDF13,14***). Where relatives’ support was lacking, they may be unaware of the importance of oral care (***TDF1***). A commonly cited enabler for oral care was if relatives supplied physical resources (A11; A45b; A52) (***TDF6,15****)*.

#### 4.3.4 Relationships

The relationships that exist (or not) between individuals (staff, residents and relatives) and external health care professionals play a role in maintaining oral health for residents and exert an influence on the oral care and service provision in the care home. Relationships were mentioned in about half of the studies (51%; n = 32) (***TDF14***).

The relationships between staff and residents are most frequently studied, highlighting factors that impact this bi-directional relationship. Factors that supported the relationship included a close bond between staff and resident (A17; A46; A58; A46; A58; A59), or similar ethnic background (A2) (***TDF7,14***). When a resident does not have confidence in a staff member’s abilities or are uncomfortable with them, then a resident may refuse assistance or support with oral care (A18; A24) (***TDF7,9,14***). Although less often studied, the relationship between care home staff and relatives is also key, relying on effective communication. When communication works well, staff and relatives work together, by sharing relevant information, identifying problems and solutions or discussing roles and responsibilities (A25; A50; A52; A53) (***TDF2,4,8***). When difficulties arise in this relationship and staff are unable to engage or involve relatives in discussion about oral health and care, this can negatively impact the resident, including delayed access to health care professionals or specialist care (A26a-c; A52; A53) (***TDF14,15***).

Peer-to-peer support between staff members is a key enabler for oral care (***TDF6,14***)**.** Informal conversations and sharing experiences about providing oral care ensure mutual staff support (A29a-d; A46; A58) (***TDF4,14***). The opportunity for these exchanges needs to be created and encouraged to enhance oral care provision (A22a-c; A46; A50; A56c) (***TDF15***). A positive team culture was associated with the ability to learn and implement new care procedures and good communication among the staff (A29a-d; A46; A50; A53; A54a-b; A56c; A57; A58) (***TDF14***). Further, there needs to be clarity among team members about staff responsibility for providing this care to reduce inconsistencies between ‘supervisory’ responsibility (usually a senior member of staff) and ‘actual’ responsibility by staff engaged in day-to-day care for residents (A6; A46; A47; A48; A50; A54a-b; A56c) (***TDF8***).

As well as relationships within the care home, the interface between care home staff and external health care professionals is also critical. Collaboration, particularly between dental professionals and care home staff, supported care for residents (A29a-d; A46; A47; A50; A53; A56b; A57) (***TDF1,14***); while a lack of collaboration limited staff access to professional advice when necessary (A40; A45b; A47; A52; A53) (***TDF14,15***). Collaboration included the use of shared language and understanding, or flexibility of health care professionals to deal with the specific issues, and circumstances for care home residents and staff. The impact of good collaboration was bidirectional: for care home staff to be advised by health care professionals to inform the care they provide for a resident; for health care professionals to have accurate information about a resident’s condition as it changes so they can advise (***TDF1,4,14***). Multi-disciplinary consultation that included care home staff and external health care professionals improved collaboration and impacted on care for residents.

### 4.4 Meso (care home) level

#### 4.4.1 Organisational structure

Broader organisational structures influence what happens in the care home, and this includes care organisation and provision (***TDF15***). Whilst not specific to oral care, these are important enablers or barriers for this area of care.

Studies reported that non-profit and newer care organisations were more supportive of staff providing oral care (A44a-c); this was not found in the for-profit sector (A27a-b) (***TDF15***). The funding structure will also have an influence on staffing arrangements in the care home. Whilst there is great variability in staffing stability across the care home sector, there are challenges with staffing that create barriers for effective oral care. This includes staff shortages (A10; A25; A29a-d; A32; A34a-b; A45a-b; A46; A47; A51; A52; A53; A54b; A57; A59), high workload (A6; A18; A26a-c; A49; A51; A55; A58), and staff turnover (A25; A28; A29a-d; A31; A46; A57) (***TDF13,15***). Staff shortages were associated with high levels of staff sickness (A11) and lack of funding in the organisation (A2; A45b; A57). When there are insufficient staff to meet care demands, then oral care provision will not be undertaken: lack of time to provide oral care was identified as a barrier (A22a-c; A45a-b; A46; A47; A49; A51; A52; A53; A54b; A56c; A58; A59) (***TDF4,15***).

#### 4.4.2 Care home management

Management attitudes and beliefs influenced oral care provision by staff in the care home with both positive and negative consequences (***TDF8,13,14***). Significantly, oral care was a lower priority for many managers compared with other aspects of care, such as ensuring adequate assistance for residents at mealtime or health and safety when helping a resident to move (A2; A45b; A46) (***TDF13,15***). For staff to be motivated, they must believe there are consequences associated with omission of oral care. However, when management perceives oral care as important and possesses knowledge of the link between oral health and general health (A29a-d), then this motivated staff to prioritise oral care in daily routines and to understand the consequences of their lack of action (***TDF1,12,14***).

Style of leadership demonstrated by the manager and senior staff is important to influence care staff to provide and embed oral care in their daily activities. This requires managers and senior staff to take time to lead with clarity and direction for staff on care that matters for residents (A6; A50; A54a-b; A56c; A57) (***TDF4,14***). This is also crucial for supporting staff with any change processes associated with implementing new oral care procedures. For example, creating time (A11), discussing the changes (A6), supporting integration of new work into existing work (A29a-d; A50; A54b) and adapting evidence to a local context (A11; A58) (***TDF1,4,15***). Involving staff in change is difficult (A6), and sustaining change takes time and effort (A44a-c; A54b; A63a-b) (***TDF4,12,15***).

#### 4.4.3 Care organisation

Systems for monitoring oral care are required to ensure its provision. System-level barriers included: managers and senior staff not recognising the importance of collecting data about residents’ oral health (A11; A45b) (***TDF1,13***); care staff not using the internal patient system to record oral care delivered (A40; A48) (***TDF4,15***); lack of analysis of routine data by management to review and improve residents’ care (A6; A48) (***TDF4,15***). In care homes committed to monitoring oral care, the following were identified as enablers: staff recognising the importance of recording oral care (A18; A50; A54b) (***TDF4,13***); using checklists (A23; A54b; A58) and sheet signing (A15) (***TDF4,15***); random oral care checks by senior staff (A23) (***TDF4,6***); and systems and processes to ensure senior staff monitor and supervise oral care by care staff (A31; A47; A50; A54b) (***TDF8,15***).

Local (care home) policies for oral care provision were highlighted as important for residents’ care. Setting clear standards of care provides clear goals for care staff. (A3; A50; A59) (***TDF4,12***). Oral care protocols were considered beneficial to guide staff practice (A25; A29a-d; A30; A33; A41; A50; A53; A59), particularly for admission processes (A8; A51), structured ongoing assessments (A31; A46; A47; A51), referral procedures (A18; A50) and support to access specialist care (A8; A50; A57) (***TDF1,4***). However, standardised protocols were sometimes perceived by staff to create an extra work burden (A35; A46) (***TDF13***). When oral care policies were absent or lacking clarity, this created a barrier for effective oral care (A3; A25; A27a-b; A29a-d; A40; A45b; A48; A52; A59), in particular for assessment process (A31; A45b), frequency of care (A31; A38), referral procedures (A29a-d; A31; A36a-b; A48) and to support staff with funding aspects of oral care (A40; A45b). Standardising a designated time slot for oral care was suggested as a strategy to support staff to provide better care (A1a-b; A58) (***TDF4,15***).

A range of benefits for residents and staff were identified when using oral care plans (A5; A8; A22a-c; A28; A40; A50; A56b; A58), checklists (A6; A23; A33; A54b; A58), manuals and booklets (A10; A11; A58), assessment instruments (A10; A22a-c; A28; A36a-b; A46; A47; A51), reminders (A22a-c; A30; A56c), visual instruction cards (A40; A58) and saliva tests (A5) (***TDF4***). Barriers related to the use of oral care tools included incorrect use (A28; A38; A47), negative perceptions of the tools (A18), lack of access (A29a-d; A46; A47) or inadequate tools to meet needs (A1a-b; A22a-c; A46; A47; A53; A54b) (***TDF5,7,9,13,15***).

Several physical resources are required by staff to provide opportunities for oral care provision (***TDF15***). There may be issues related to the availability and accessibility of these resources. When not available or not adequate, this will create a barrier for staff. Examples of relevant physical resources includes toothbrush, toothpaste, and mouthwash (e.g., A18, A22a-c, A41; A45b; A53; A56b). More specialist equipment, such as an ultrasonic denture cleaning device was mentioned in one study (A4a-b). Lack of denture labelling was also a concern (A3). Physical resources/facilities influencing care included the availability of a dental chair (A38), adequate space (in bathrooms), and lighting (A40; A54b). Finally, information and communication technology used to access relevant information about a resident were also identified as an important physical resource to promote care (A29a-d; A50) (***TDF1,15***).

Diet and nutrition are important factors that influence an individual’s oral health. Whilst oral health status of residents is considered when planning menus and meal provision (A8; A56a,b) (***TDF1,4***), a high proportion of care homes provide regular sugary snacks for residents as part of everyday care (A14; A46; A54b; A56b) (***TDF15***).

#### 4.4.4 Oral care training for care home staff

Opportunities for oral care training were linked to staff capabilities and reported as a barrier or enabler to oral care provision in almost three-quarters of the included studies (71%; n = 45) (***TDF1,15***). Lack of time to complete training in working hours was often cited as a barrier (***TDF15***). Non-compulsory and free training, had a mixed reaction from staff: sometimes welcomed (A10) but not by all (A3) (***TDF13***).

The content, delivery and format, frequency and accessibility for care staff are important considerations for training interventions. A variety of oral care topics were highlighted but more specific topics on how to support people with dementia (A3; A51; A58; A59), or oral cancer (A26a-c; A45a) were highlighted (***TDF1***). ‘Hands-on’ opportunities for learning (or ‘on the job’ training), practical tips and on-site training (as opposed to travelling to other locations) were considered particularly helpful (A10; A32; A50; A58) (***TDF5,15***). Provision of different modes of delivery were important, with a combination of these modes advocated (A31; A50). More specifically, online training was reported as particularly suitable for staff working unsocial hours, as it gave them more flexibility to choose when to do the training (A10; A50). However, online training was sometimes difficult because of problems with accessibility and volume of learning materials (A32). Alternative mechanisms to support staff capabilities were identified: ‘in-house’ coaching aimed to support staff to carry out oral care with residents alongside more experienced colleagues (for example registered nurses or oral care champions) was viewed as an enabler (A32; A46; A50) (***TDF14,15***). Oral health champions (a staff member allocated to a specialist role to lead, co-ordinate and advise on oral care in the care home) were identified as a key enabler (A41; A47; A50; A57; A58) to support staff, promote opportunities and raise awareness about this important aspect in everyday care (***TDF8,14,15***).

Physical resources to support training could create barriers, for example limited access to electronic equipment (A3; A30), a suitable venue (A13), or lack of paper materials (A22a-c) (***TDF15***). All studies highlighted the importance of regular and frequent training opportunities regardless of delivery or format.

### 4.5 Macro (policy) level

#### 4.5.1 National or regional policy

National or regional policies for oral care provision were important for residents’ care. Some studies focused on the benefit of having policies in place (A3; A27a-b; A47; A57; A59), particularly when these provide guidance for challenging requirements, residents’ circumstances or the care home environment (A22a-c; A58) (***TDF1,15***). National training programs (rather than organisational) were advocated as important enablers for oral care provision (A10; A11; A45b; A52). However, other studies pointed out the difficulties of national or regional policy, including the use of patronising language in the guidelines (A22a-c), the lack of long-term and stable vision, and poor integration with other guidelines (A29a-d; A45b; A52; A58) (***TDF13,15***). Monitoring care provision in care homes at a regional or national level was considered an enabler (A3; A47) (***TDF4,6***). This requires policies that are enacted, and which guarantee regular checks of the oral care provided in care homes.

The attitudes and beliefs of policy makers may act as a barrier, especially where there is a belief that oral care is ‘basic’ care and a lack of understanding of the complexities associated with this population and the impact of oral care on oral health and general health (A29a-d; A45b; A52) (***TDF1,13,14***). This generates challenges for care staff and residents to be able to access relevant resources. Lack of access and the funding to access specialist dental care are significant barriers (A26a-c; A45b; A53; A57) (***TDF15***). The provision of additional funding was an enabler, for example, funding to support dental staff to provide dedicated oral care and treatment for care home residents (A44a-c; A47; A57) (***TDF6,15***).

## 5 External health care professionals

There were a range of barriers and enablers related to health care professionals who support (or could support) residents (and their relatives) in care homes and the staff providing oral care.

Residents’ access to specialist dental care inside or outside the care home was identified by several studies as a major concern (A3; A8; A14; A24; A26a-c; A31; A34a-b; A38;, A40; A45b; A52; A53; A54a; A57) (***TDF13,15***). Some studies identified ways of improving access including: domiciliary dental care services (A57); specific arrangements for specialist care with the care organisation (A8); a dental care referral pathway (A3; A50); and support within the care home from dental students (A26a-c; A31) or dental hygienists (A37; A46; A47) (***TDF14,15***). Dental specialists can offer support for care home staff (A6; A29a-d) (***TDF1,14***). This support may take different forms, from flexible one-to-one advice (A11) to case conferences for individual residents (A12) (***TDF4***). Further, specialists working with health and social care professionals involved with the resident’s care will benefit the residents and staff (A26c; A47; A53) (***TDF14,15***).

The attitude and beliefs of oral care professionals can be a barrier: where the professional perceives care in this setting a lower priority than other demands on their time then this will limit access to services for these individuals (A26a-c) (***TDF8,13***). For those engaging with care home residents and staff, communication skills were critical for the oral care professional to establish a relationship with and gain trust of the resident (A24) (***TDF2,14***). The benefit of involving dental and nursing students with care home residents was highlighted (A27a-b) as helping to shape the attitudes and beliefs of future professionals about this aspect of care (***TDF1,13,14***).

## 6 Discussion

This scoping review ‘takes stock’ of the evidence on enablers and barriers influencing oral care support for older people living in care homes with their oral health and care needs. Its novel application of the COM-B and TDF frameworks has enabled a structured analysis of the behavioural components influencing staff practices and revealed a complex interplay of factors. While oral health is increasingly recognised as vital to older people’s wellbeing and quality of life, the review highlights the multi-layered influences on oral care in long-term care settings, spanning micro, *meso* and macro-levels. The interactions between these levels compound enablers and barriers to oral care in this context.

Staff attitudes, emotional responses and perceived responsibility for oral care were identified as central to the support of residents in long-term care settings. Resident engagement with, and their ability to communicate, oral care needs to staff influenced outcomes. The relationship between staff and residents was recognised as important, with trust and familiarity enabling more effective oral care support. Relatives, when informed and involved in care, played a facilitative role, as advocates or securing resources and/ or access to dental services. Peer support between long-term care staff, and with external health care professionals, improved care planning and responsiveness. Our review highlights that oral care interventions in this context need to address more than technical skill and competence. Relational dynamics and the emotional labour of oral care ‘work’ are fundamental considerations for any future interventions.

The review highlighted a high volume of studies on the effectiveness of oral care training interventions for long-term care staff. Training effectiveness depends on relevance, accessibility and delivery format. Staff valued practical, hands-on learning, particularly on-site or ‘on the job’ training. Mentorship from experienced colleagues or oral health champions, were identified as key enablers: supporting embedding oral care into daily routines and enabling staff confidence. Several recent studies also highlighted the importance of formally designated oral health champions or liaison staff who acted as local leaders, educators and points of contact between care homes and dental services. These roles appeared particularly valuable for sustaining implementation efforts and maintaining oral health as a visible organisational priority. Together these findings underscore the need for regular, accessible, and context-sensitive training that is supported by leadership and organisational infrastructure. More recent studies suggest that successful implementation of oral care practices depends on organisational culture, with oral care more likely to be sustained in settings where managers and teams collectively regarded oral health as a routine and valued component of care.

Organisational structures and management practices significantly shaped the conditions under which oral care was delivered. Staffing pressures (including shortages and high turnover), leadership attitudes and resource constraints were associated with prioritisation of oral care for residents in this context. The variation (and sometimes absence of) protocols and systems for monitoring oral care influenced staff accountability and the consistency of its provision for residents. Conversely, studies evaluating structured assessment and monitoring systems demonstrated improvements in staff awareness, documentation, accountability and oral-health prioritisation, suggesting that routine assessment frameworks may be important mechanisms for sustaining practice change. Clearly, embedding oral care into routine care practices in long-term care settings demands supportive leadership, adequate resources and integrated systems. More recent studies suggest that oral care challenges are not solely attributable to individual staff behaviours but frequently reflect wider implementation failures within care home systems. The findings therefore position oral health as an organisational quality and safety issue requiring system-level solutions rather than relying exclusively on frontline staff behaviours.

The updated search (2022 to 2026) allowed consideration of post-pandemic evidence. Staff reported increased workload, workforce shortages and anxiety, while residents experienced reduced family contact, diminished social engagement and declines in physical and psychological wellbeing. The pandemic also increased staff awareness of infection-control practices during oral care and may have increased resident dependency and reduced opportunities for oral-health support. Although some contextual changes were apparent, including workforce pressures, staffing shortages and altered patterns of resident dependency, the principal barriers and enablers identified remained consistent with those reported in studies conducted before the pandemic. This provides reassurance that the conceptual framework developed from the review remains relevant across these different time periods.

However, future oral-health interventions should be sensitive to the longer-term effects of pandemic-related changes within care-home environments.

National and regional policies and funding mechanisms play a critical role in shaping the conditions under which oral care is delivered, as well as residents’ access to specialist support. Studies highlighted limitations in existing policies, including patronising language, poor integration with other guidelines, and a lack of long-term strategic vision for oral care provision in long-term care. Our review highlights the need for integrated, well-resourced policies and professional engagement to embed oral health as a core component of care for older people living in long-term residential care. The newer evidence further demonstrates that successful oral care is strongly dependent upon interprofessional collaboration between care home staff, dental professionals, managers and wider health services. In several studies, structured partnerships with dental hygienists, domiciliary dental teams and oral-health educators improved awareness, assessment, referral processes and continuity of care.

### 6.1 Implications for policy, practice and research

There are several important implications for policy and practice arising from this review.

National oral health standards, aligned with national guidance, are needed to ensure consistent and accountable care. Oral health assessments and care planning could be better integrated into country-specific regulatory or quality frameworks. There is an urgent need to improve residents’ access to dental services. Training programmes tailored to the needs of long-term care staff, including dementia-specific oral care and practical skills are needed. Emerging evidence also highlights the potential value of domiciliary dental care models for improving access among frail and cognitively impaired residents, although sustainable funding mechanisms and workforce availability remain important challenges. But importantly, addressing these policy implications demands the involvement of relevant stakeholders, including staff, residents, relatives, and dental professionals in any policy development.

In practice, individual staff require enhanced knowledge and skills, supportive working conditions and reinforcement of positive attitudes and beliefs towards oral care. Oral care needs to be embedded as part of essential personal care using structured protocols and care plans, ensuring ready access to appropriate oral care supplies, allocating protected time for mouth care within daily routines, and ensuring that staffing levels and skill mix allow staff to complete oral care without compromising other duties. The introduction of oral health champions in the care setting can act as role models, leading, supporting, and monitoring oral care practices. Facilitating regular communication within teams about oral care challenges may also reduce the emotional labour and isolation that staff experience when providing care to residents who are distressed or resist mouth care. Regular, accessible oral care training using blended formats (e.g., online, hands-on, peer coaching), supported by leadership, is required. Collaborative relationships with external dental professionals need to be fostered to improve continuity and responsiveness. Finally, improving documentation and data use will facilitate the monitoring of oral health outcomes and inform continuous improvement, fostering staff ownership and accountability.

Building on the scoping review findings and the COMMIT programme of research, evidence- and theory-informed guiding principles have been developed to support care home, staff and partner organisations in strengthening oral care provision and embedding oral health within everyday care practices (Devi et al., 2026). Our theoretically informed review also supports the development of a multi-component, context-sensitive intervention. For example, targeted interventions that address ‘beliefs about consequences’ to enhance staff motivation, or ‘environmental context and resources’ to improve opportunities for staff. Ensuring such an intervention is underpinned by behavioural science offers a lens to identify and address behavioural determinants in any planned intervention. Future research should focus on designing and testing multi-component interventions informed by COM-B and TDF, with attention to feasibility, acceptability, and sustainability in long-term care home settings.

### 6.2 Strengths and limitations

There are strengths and limitations of our review and its findings.

The review synthesises evidence from varied perspectives (including residents and relatives) across micro (individual), *meso* (organisational), and macro (policy/system) levels, offering a comprehensive understanding of the factors influencing oral care in long-term residential care settings. We have attempted to balance clarity with the level of detail required to represent the breadth of evidence synthesised across these multi-level factors.

The review goes beyond listing barriers and enablers by showing how factors at different levels interact and compound challenges or support for oral care. The use of the COM-B model and TDF domains provides a structured and behaviourally informed lens to understand barriers and enablers. The findings are highly applicable to real-world care settings, with clear implications for service delivery, staff training, leadership and policy development. The review highlights the importance of interpersonal and interprofessional relationships, which are often underexplored in oral health literature.

Our searches did not impose language restrictions, however only English-language studies were included. This may have resulted in the exclusion of relevant evidence published in other languages. The included studies represent a range of geographical and contextual settings; however, most were conducted in high income countries. This imbalance reflects the distribution of available published research in English rather than deliberate exclusion of lower- or middle-income contexts. Nevertheless, the review included studies from a wide range of countries and healthcare systems, including Malaysia, China, India, Iran, Brazil, Chile and South Africa. Across these diverse settings, common influences on oral care provision were evident, including workforce pressures, competing care priorities, limited access to oral health expertise, resource constraints and the importance of supportive organisational cultures and leadership. While the consistency of these findings suggests that several barriers and enablers may be transferable across socio-economic settings, their manifestation and relative importance are likely to be influenced by local care infrastructures, funding arrangements and workforce models. Further research from low- and middle-income countries is needed to strengthen understanding of these contextual differences and identify setting-specific solutions. We have provided detailed information about the included literature to enable the reader to assess the relevance and limitations of the findings within their own context.

We recognise that categorising all adults aged 65 years and older as ‘older people’ may introduce age-related bias. The needs and levels of dependency of those aged 65–70 years can differ substantially from those aged 80 years and over, who are more likely to experience frailty, cognitive impairment, and multimorbidity. These differences may influence both the nature of oral care required and the challenges faced by care staff. However, because our aim was to map the breadth of available evidence, and because definitions of ‘older people’ vary internationally, we adopted an inclusive approach consistent with scoping review methodology. Future research could examine differences between age subgroups and dependency levels within the older population.

The consistency of findings across both searches suggests that the key enablers and barriers to oral care provision in long-term care have remained stable over time. To support transparency and future replication, the full search strategy is provided as a supplementary file. To our knowledge, no other review has addressed this topic using the same theoretical framework, further supporting the originality and relevance of the review. Stakeholder consultations also confirmed that the findings aligned closely with their experiences and understanding of oral care provision in long-term care settings (Vinall-Collier et al., 2026).

The included studies vary in methodological quality. Studies were not excluded based on quality criteria because of the scoping review method, but this may affect the strength of the conclusions drawn. While the review identifies potential interventions, we did not aim to assess their feasibility, cost-effectiveness, or sustainability in practice. We noted a gap in theory-informed research in this area. However, we have used theory to synthesise our review of enablers and barriers for oral care provision in long-term residential care settings.

## 7 Conclusion

Oral care provision in long-term care settings is influenced by a complex interplay of factors. We have identified and synthesised these factors at the individual (micro), organisational (*meso*) and wider social and political (macro) levels. This highlights that attributing poor oral care for older people living in care homes solely to care staff is inappropriate. While staff shortages, workload pressures and limited training influence the delivery of oral care, the time that staff dedicate to oral care or their knowledge and skills must be understood within a broader context.

Use of the COM-B model and TDF identified that the majority of barriers are in the opportunity domain: the environmental context, lack of resources available to care home staff, alongside the social environment are the biggest barriers to providing oral care in this setting. Interventions aimed at improving oral care must go beyond technical training to address the emotional labour involved, the importance of relational dynamics and the structural conditions and systemic constraints under which care is delivered. In practice, this includes embedding oral care into daily routines, ensuring ready access to appropriate supplies, allocating protected time for mouth care, and supporting positive social influences through oral health champions, regular team communication, and ongoing training. Strengthening collaboration with dental professionals and improving documentation and monitoring processes can further enable consistent, compassionate, and accountable oral care. The COM-B and TDF frameworks offer a robust foundation for designing multi-component, context-sensitive interventions that appreciate the realities of long-term care settings.

## Ethics declaration

The author(s) declare(s) that the study does not involve humans nor animal subjects.

## Disclaimer

The views expressed are those of the authors and not necessarily those of the NIHR or the Department of Health and Social Care.

## Funding acknowledgement

This study was funded by the 10.13039/501100000272National Institute for Health and Care Research (NIHR) Health and Social Care Delivery Research programme (NIHR131506).

## CRediT authorship contribution statement

**Karen Spilsbury:** Writing – original draft, Supervision, Methodology, Funding acquisition, Formal analysis, Conceptualization. **Julia Csikar:** Writing – review & editing, Investigation, Funding acquisition, Formal analysis, Conceptualization. **Reena Devi:** Writing – review & editing, Methodology, Investigation, Funding acquisition, Formal analysis, Conceptualization. **Karen Vinall-Collier:** Writing – review & editing, Methodology, Investigation, Funding acquisition, Formal analysis, Conceptualization. **Magda Jordao:** Writing – review & editing, Investigation, Formal analysis. **Judy Wright:** Writing – review & editing, Resources, Methodology, Investigation, Funding acquisition, Conceptualization. **Carl Thompson:** Formal analysis, Investigation. **Alys Wyn Griffiths:** Writing – review & editing, Funding acquisition, Conceptualization. **Paul Wilson:** Writing – review & editing, Supervision, Funding acquisition, Conceptualization. **Edna Feenan:** Writing – review & editing, Investigation, Funding acquisition. **Karen Winterburn:** Writing – review & editing, Supervision, Investigation. **Sharon Earnshaw:** Writing – review & editing, Supervision, Investigation. **Gail V.A. Douglas:** Writing – review & editing, Supervision, Methodology, Investigation, Funding acquisition, Conceptualization.

## Declaration of competing interest

The authors declare the following financial interests/personal relationships which may be considered as potential competing interests: Karen Spilsbury reports financial support was provided by National Institute for Health and Care Research. Karen Spilsbury reports a relationship with National Institute for Health and Care Research that includes: funding grants. If there are other authors, they declare that they have no known competing financial interests or personal relationships that could have appeared to influence the work reported in this paper.

## Appendix 1 Included studies

A1a. Acosta JM, Coe J, Carrico C. Long-term Care Facility Care Providers’ Confidence in Assessing Oral Health. 2017 IADR/AADR/CADR General Session (San Francisco, California); 03/24/2017; San Francisco, California2017.

A1b. Acosta JM, Coe J, Carrico C. Oral health confidence in long-term care facilities­­. Virginia Dental Journal. 2018;95(4):34–41.

A2. Adebayo B, Durey A, Slack-Smith LM. Culturally and linguistically diverse (CALD) carers' perceptions of oral care in residential aged care settings in Perth, Western Australia. Gerodontology. 2017;34(1):33–41.

A3. Ahmad B, Landes D, Moffatt S. Dental Public Health In Action: Barriers to oral healthcare provision for older people in residential and nursing care homes: A mixed method evaluation and strategy development in County Durham, North East England. Community Dent Health. 2018;35(3):136–9.

A4a. Schwindling FS, Krisam J, Hassel AJ, Rammelsberg P, Zenthöfer A. Long-term success of oral health intervention among care-dependent institutionalized seniors: Findings from a controlled clinical trial. Community Dent Oral Epidemiol. 2018;46(2):109–17.

A4b. Deisenhofer U, Baumgart D, Cabrera T, Hassel A, Zenthöfer A, Rammelsberg P. Effectiveness of an Oral Health Intervention for Institutionalized Elderly. 2016 IADR/APR General Session (Seoul, Korea); 06/25/2016; Seoul, Korea2016.

A5. Deutsch A, Siegel E, Cations M, Wright C, Naganathan V, Brodaty H. A pilot study on the feasibility of training nurses to formulate multicomponent oral health interventions in a residential aged care facility. Gerodontology. 2017;34(4):469–78.

A6. Eldh AC, Olai L, Jönsson B, Wallin L, Denti L, Elf M. Supporting first-line managers in implementing oral care guidelines in nursing homes. Nordic Journal of Nursing Research. 2018;38(2):87–95.

A7 Godoy J, Rosales E, Garrido-Urrutia C. Oral health care related beliefs among caregivers of institutionalized elderly in Antofagasta city, Chile, 2019. ODONTOESTOMATOLOGIA. 2021;23(38).

A8. Gopalakrishnan A, Kahu E, Jones L, Brunton P. Access and barriers to oral health care for dependent elderly people living in rest homes. Gerodontology. 2019;36(2):149–55.

A9. Hiltunen K, Fogelholm N, Saarela RKT, Mantyla P. Survey of health care personnel's attitudes toward oral hygiene in long-term care facilities in Finland. Spec Care Dentist. 2019;39(6):557–63.

A10. Hoang H, Barnett T, Maine G, Crocombe L. Aged care staff's experiences of 'Better Oral Health in Residential Care Training': A qualitative study. Contemporary Nurse. 2018;54(3):268–83.

A11. Howells EP, Davies R, Jones V, Morgan MZ. Gwen am Byth: a programme introduced to improve the oral health of older people living in care homes in Wales - from anecdote, through policy into action. Br Dent J. 2020;229(12):793–9.

A12. Huynh J, Donnelly LR, Brondani MA. Exploring dental student participation in interdisciplinary care team conferences in long-term care. Gerodontology. 2017;34(2):249–56.

A13. Ildarabadi EH, Armat MR, Motamedosanaye V, Ghanei F. Effect of Oral Health Care Program on Oral Health Status of Elderly People Living in Nursing Homes: a Quasi-experimental Study. Mater Sociomed. 2017;29(4):263–7.

A14. Jamsa H, Laitala ML, Vahanikkila H, Syrjala AM. Implementation of oral hygiene practices in nursing homes - the view of supervisor nurses. Acta Odontol Scand. 2021:1–7.

A15. Johansson I, Torge CJ, Lindmark U. Is an oral health coaching programme a way to sustain oral health for elderly people in nursing homes? A feasibility study. Int. 2020;18(1):107–15.

A16a. Johnson I, Jones R, Morgan M. Oral Care, Loss of Identity and Dignity in Care Homes. 2017 IADR/AADR/CADR General Session (San Francisco, California); San Francisco, California2017.

A16b. Johnson IG, Morgan MZ, Jones RJ. Oral care, loss of personal identity and dignity in residential care homes. Gerodontology. 2022.

A17. Jones RJ, Johnson IG, Morgan MZ. Family and friends: Supporting oral care in care homes. Gerodontology. 2019;36(3):258–66.

A18. Keboa M, Beaudin A, Cyr J, Decoste J, Power F, Hovey R, et al. Dentistry and nursing working together to improve oral health care in a long-term care facility. Geriatr Nurs. 2019;40(2):197–204.

A19. Kim B, Lee KH, Cho E, Kim H. Behaviors of care providers and resistiveness to oral care of persons living with dementia. Geriatr Nurs. 2021;42(4):915–8.

A20. Klotz AL, Zajac M, Ehret J, Hassel AJ, Rammelsberg P, Zenthofer A. Development of a German version of the Oral Health Assessment Tool. Aging Clin Exp Res. 2020;32(1):165–72.

A21a. Kohli R, Nelson S, Ulrich S, Finch T, Hall K, Schwarz E. Dental care practices and oral health training for professional caregivers in long-term care facilities: An interdisciplinary approach to address oral health disparities. Geriatr Nurs. 2017;38(4):296–301.

A21b. Schwarz E, Kohli R, Rosales J, Nelson S. Dental Care Practices and Attitudes of Professional Caregivers Towards Seniors in Oregon. 2015 IADR/AADR/CADR General Session (Boston, Massachusetts); 03/14/2015; Boston, Massachusetts2015.

A22a. Langley J, Wassall R, Verey A, Geddis-regan A, Brocklehurst P, McKenna G, et al. Co-design: A Route to Utilisation of Research in Care Homes?

Exploration of key Oral Health Outcomes for Dependent Older Adults. 2020 IADR/AADR/CADR General Session (Washington, DC, USA); 07/22/2021; Washington, D.C., USA2020.

A22b. Wassall R, Teal G, Valentine L, Wilson R, Moynihan P. Integrating Evidence Using Co-Design to Develop Complex Interventions in Dentistry. 2018 IADR/PER General Session (London, England); 07/27/2018; London, England2018.

A22c. Langley J, Wassall R, Geddis-Regan A, Watson S, Verey A, McKenna G, et al. Putting guidelines into practice: Using co-design to develop a complex intervention based on NG48 to enable care staff to provide daily oral care to older people living in care homes. Gerodontology. 2022.

A23. Laurence B, Rogers I, Grant-Mills D, Smith D, Tefera E, Barbarinde A, et al. Reducing Dental Plaque Scores in Long-term Care Facilities Using a Checklist and Random Inspections: A Pilot Study. Bull Tokyo Dent Coll. 2019;60(3):177–84.

A24. Letchumanan D, Mohamad Norpi N, Yusof ZYM, Razak IA, Abu Kasim NH, Abdullah NA, et al. Caregivers' perceptions towards oral healthcare services for elders living in Malaysian nursing homes-A qualitative study. Gerodontology. 2020;37(4):332–41.

A25. Lowman SG, Zimmerman S, Ward K, Weintraub JA. Family perceptions of mouth care for assisted living residents with dementia: Implications for care. Geriatr Nurs. 2021;42(4):926–34.

A26a. Maramaldi P, Cadet T, Burke SL, LeCloux M, White E, Kalenderian E, et al. Oral health and cancer screening in long-term care nursing facilities: Motivation and opportunity as intervention targets. Gerodontology. 2018;35(4):407–16.

A26b. Maramaldi P, Cadet T, Burke S, LeCloux M, White E, Kinnunen TH, et al. Administrators' Perceptions of Oral Health Care and Cancer Screening in Long-Term Care Nursing Facilities. J Appl Gerontol. 2019;38(10):1391–420.

A26c. Maramaldi P, Cadet T. Barriers to providing oral health and oral cancer screening in long-term care facilities. Psycho-Oncology. 2014;1):76.

A27a. Marin Zuluaga DJ, Gil Montoya JA. Institutionalised Elderly' Oral Health, After a Carers Oral Care Training-Program. 2011 IADR/AADR/CADR General Session (San Diego, California); 03/18/2011; San Diego, California2011.

A27b. Montoya JAG, Zuluaga DJM, Willumsen T. Effectiveness of a training program for the nursing staff on the oral health of institutionalised aged. Randomised trial. Acta Odontológica Colombiana. 2019;9(1):58–70.

A28. McConnell ES, Lee KH, Galkowski L, Downey C, Spainhour MV, Horwitz R. Improving Oral Hygiene for Veterans With Dementia in Residential Long-term Care. J Nurs Care Qual. 2018;33(3):229–37.

A29a. Niesten D, Gerritsen AE, Leve V. [What promotes and hinders integration of oral care in general care? Part 2. Policy, organization, implementation]. Ned Tijdschr Tandheelkd. 2021;128(10):495–502.

A29b. Niesten D, Gerritsen AE, Leve V. [What promotes and hinders integration of oral care in general care? Part 1. Vision, attitude and culture]. Ned Tijdschr Tandheelkd. 2021;128(10):485–94.

A29c. Niesten D, Gerritsen AE, Leve V. Barriers and Facilitators to integrate Oral Health care for Older Adults in General (Basic) Care in East Netherlands. Part 2 Functional Integration. Gerodontology. 2021;38(3):289–99.

A29d. Niesten D, Gerritsen AE, Leve V. Barriers and facilitators to integrate oral health care for older adults in general (basic) care in East Netherlands. Part 1: Normative integration. Gerodontology. 2021;38(2):154–65.

A30. Patel R, Robertson C, Gallagher JE. Collaborating for oral health in support of vulnerable older people: co-production of oral health training in care homes. J Public Health (Oxf). 2019;41(1):164–9.

A31. Patterson Norrie T, Villarosa AR, Kong AC, Clark S, Macdonald S, Srinivas R, et al. Oral health in residential aged care: Perceptions of nurses and management staff. Nurs. 2020;7(2):536–46.

A32. Pawluk DL, Dale C, Lai JY, Villacorta-Siegal N, Smith J, Joseph K, et al. The impact of oral health education taken by personal support workers caring for a geriatric population in a long-term care facility: A mixed-methods study. Gerodontology. 2021;38(4):373–86.

A33. Red A, O'Neal PV. Implementation of an Evidence-Based Oral Care Protocol to Improve the Delivery of Mouth Care in Nursing Home Residents. J Gerontol Nurs. 2020;46(5):33–9.

A34a. Sigurdardottir AS, Geirsdottir OG, Ramel A, Arnadottir IB. Cross-sectional study of oral health care service, oral health beliefs and oral health care education of caregivers in nursing homes. Geriatr Nurs. 2022;43:138–45.

A34b. Sigurdardottir A, Arnadottir I, Holbrook WP. Oral Care in a Icelandic Nursing home. 2017 Continental European and Scandinavian Divisions Meeting (Vienna, Austria); 09/21/2017; Vienna, Austria2017.

A35. Torres de Medeiros Alves AK, Alves Esmeraldo C, de Carvalho Costa MS, Padilha Honório ML, de Araújo Nunes VM, Lessa de Freitas AA, et al. Ações desenvolvidas por cuidadores de idosos institucionalizados no Brasil. Avances en Enfermeria. 2018;36(3):273–82.

A36a. Shibata S, Ito K, Stegaroiu R, Tsukada S, Ohuchi A. Utilization of an oral screening sheet by nurses and caregivers in an elderly nursing home: comparison with a dentist as the reference. 2014 Japanese Division Meeting (Osaka, Japan); Osaka, Japan2014.

A36b. Tsukada S, Ito K, Stegaroiu R, Shibata S, Ohuchi A. An oral health and function screening tool for nursing personnel of long-term care facilities to identify the need for dentist referral without preliminary training. Gerodontology. 2017;34(2):232–9.

A37. Urata JY, Couch ET, Walsh MM, Rowe DJ. Nursing Administrators' Views on Oral Health in Long-Term Care Facilities: An exploratory study. J Dent Hyg. 2018;92(2):22–30.

A38. Villarosa AR, Clark S, Villarosa AC, Norrie TP, Macdonald S, Anlezark J, et al. Promoting oral health care among people living in residential aged care facilities: Perceptions of care staff. Gerodontology. 2018;35(3):177–84.

A39. Volk L, Spock M, Sloane PD, Zimmerman S. Improving Evidence-Based Oral Health of Nursing Home Residents Through Coaching by Dental Hygienists. J Am Med Dir Assoc. 2020;21(2):281–3.

A40. Weening-Verbree LF, Schuller DAA, Cheung SL, Zuidema P, Schans P, Hobbelen D. Barriers and facilitators of oral health care experienced by nursing home staff. Geriatr Nurs. 2021;42(4):799–805.

A41. White BA, Van Haitsma K, Lauver L, Sillner A. Improving Uptake of Evidence-Based Oral Care in Long-Term Care. Annals of Long Term Care. 2019;27(9):e6-e14.

A42. Yanagisawa S, Yoshioka M, Shirayama Y. Survey on Nursing Home Caregivers' Basic Knowledge of Oral Health Management: Dental Terminology. Dent J (Basel). 2018;6(3):02.

A43. Zhang J, Wang Z, Li Y, Yu P, Cao X, Xu X, et al. Effects of a Caregiver Training Program on Oral Hygiene of Alzheimer's Patients in Institutional Care. J Am Med Dir Assoc. 2021;22(7):1429–34.e1.

A44a. Zimmerman S, Sloane PD, Ward K, Wretman CJ, Stearns SC, Poole P, et al. Effectiveness of a Mouth Care Program Provided by Nursing Home Staff vs Standard Care on Reducing Pneumonia Incidence: A Cluster Randomized Trial. JAMA netw. 2020;3(6):e204321.

A44b. Wretman CJ, Zimmerman S, Ward K, Sloane PD. Measuring Self-Efficacy and Attitudes for Providing Mouth Care in Nursing Homes. J Am Med Dir Assoc. 2020;21(9):1316–21.

A44c. Zimmerman S, Wretman CJ, Ward K, Tandan M, Sloane PD, Preisser JS. Fidelity and sustainability of Mouth Care Without a Battle and lessons for other innovations in care. Geriatr Nurs. 2020;41(6):878–84.

A45a Balwanth, S. and Singh, S. (2023) ‘Caregivers’ knowledge, attitudes, and oral health practices at long-term care facilities in KwaZulu-Natal’, *Health SA Gesondheid*, 28, pp. 1–9. https://doi.org/10.4102/hsag.v28i0.2147

A45b Balwanth, S. and Singh, S. (2023) ‘Oral healthcare provision at long-term care facilities in eThekwini: Perspectives of coordinators’, *African Journal of Primary Health Care & Family Medicine*, 15(1), pp. e1-e10. https://doi.org/10.4102/phcfm.v15i1.3884

A46 Bellander, L., Angelini, E., Andersson, P., Hägglin, C. and Wijk, H. (2024) ‘A preventive care approach for oral health in nursing homes: a qualitative study of healthcare workers’ experiences’, *BMC Geriatrics*, 24(1), p. 803. https://doi.org/10.1186/s12877...024...05396...1

A47 Bellander, L., Wijk, H., Andersson, P. and Hägglin, C. (2025) ‘Oral health care for frail older adults in nursing homes from a management perspective: a survey-based study’, *BMC Oral Health*, 25(1). https://doi.org/10.1186/s12903...025...07327-x

A48 Brodie, A., Caspar, S., Hopper, T., Compton, S. and Slaughter, S. (2025) ‘Exploring oral care in long-term care homes: an institutional ethnography’, *Special Care in Dentistry*, 45(3), p. e70062. https://doi.org/10.1111/scd.70062

A49 Chen, M.X., Weng, Y.Q., Zhang, J.W., Gu, L.Y., Chen, W.Y., Qiao, M.T., Wang, M.D., Huang, X.R., Chen, L. and Zhang, L.J. (2023) ‘Factors associated with nurses’ attitudes for providing oral care in geriatric care facilities: a cross-sectional study’, *BMC Oral Health*, 23(1). https://doi.org/10.1186/s12903...023...03517...7

A50 Crittenden, J.A., Nelligan, L.I., O’Connell, D. and Brennan, L. (2024) ‘Educating long-term care staff on older adult oral health: Maine’s oral team-based initiative vital access to education (MOTIVATE) program’, *Journal of Public Health Dentistry*, 84(3), pp. 334–339. https://doi.org/10.1111/jphd.12630

A51 Dumbuya, J., Marwaha, R.S., Shah, P.K. and Challa, S. (2024) ‘To assess the knowledge, attitudes, and confidence of caregivers and administrators towards the oral health of nursing home residents in San Antonio, Texas’, *BMC Geriatrics*, 24(1), p. 511. https://doi.org/10.1186/s12877...024...04784-x

A52 Fazli, F.H.M., Nordin, N., Mohamad, M.S.F., Khan, A.J. and Ahmad, M.S. (2024) ‘Exploring barriers to oral healthcare at private residential aged care facilities in Selangor, Malaysia: a pilot study’, *Discover Public Health*, 21(1), pp. 1–13. https://doi.org/10.1186/s12982...024...00296...6

A53 Gomez-Rossi, J., Schwartzkopff, J., Muller, A., Hertrampf, K., Abraham, J., Gassmann, G., Schlattmann, P., Gostemeyer, G. and Schwendicke, F. (2022) ‘Health policy analysis on barriers and facilitators for better oral health in German care homes: a qualitative study’, *BMJ Open*, 12(3), p. e049306. https://doi.org/10.1136/bmjopen-2021...049306

A54a Hendry, A., Baker, S.R., McKenna, G., Tsakos, G., Chestnutt, I., Smith, C., Jones, V., O’Neill, C., Jenkins, A., Evans, R., Syed, S.S., Mirza, A., Harvey, M., Karki, A., Moons, K., Sandom, F., Donaldson, M., Lappin, C., Shepherd, K. and Brocklehurst, P.R. (2024) ‘Setting the context for a complex dental intervention of role substitution in care homes: Initial process evaluation findings’, *Gerodontology*, 41(4), pp. 535–546. https://doi.org/10.1111/ger.12749

A54b Hendry, A., Baker, S.R., McKenna, G., Tsakos, G., Chestnutt, I., Smith, C., O’Neill, C., Jenkins, A., Evans, R., Syed, S.S., Harvey, M., Karki, A., Moons, K., Sandom, F., Donaldson, M., Lappin, C., Shepherd, K., Morgan, L. and Brocklehurst, P.R. (2026) ‘Using role substitution to improve oral health in care homes: a process evaluation’, *Gerodontology*, 43(1), pp. 36–43. https://doi.org/10.1111/ger.70012

A55 Hidaka, R., Matsuo, K., Maruyama, T., Kawasaki, K., Tasaka, I., Arai, M., Sakoda, S., Higuchi, K., Jinno, E., Yamada, T. and Minakuchi, S. (2023) ‘Impact of COVID-19 on the surrounding environment of nursing home residents and attitudes towards infection control and oral health care among nursing home staff in Japan’, *Journal of Clinical Medicine*, 12(5), p. 1944. https://doi.org/10.3390/jcm12051944

A56a Jämsä, H., Laitala, M.L., Vähäniikkilä, H. and Syrjälä, A.M. (2022) ‘Implementation of oral hygiene practices in nursing homes: the view of supervisor nurses’, *Acta Odontologica Scandinavica*, 80(4), pp. 308–314. https://doi.org/10.1080/00016357.2021.2009026

A56b Jämsä, H., Laitala, M.L., Kaakinen, P., Ylöstalo, P. and Syrjälä, A.M. (2024) ‘Implementation of oral hygiene practices in nursing homes: a qualitative approach among supervisor nurses’, *Acta Odontologica Scandinavica*, 82(1), pp. 25–32. https://doi.org/10.1080/00016357.2023.2252493

A56c Jämsä, H., Laitala, M.L., Kaakinen, P., Ylöstalo, P. and Syrjälä, A.M. (2024) ‘The antecedents of oral care in nursing facilities: a qualitative interview study among supervisor nurses’, *Acta Odontologica Scandinavica*, 83, pp. 302–307. https://doi.org/10.2340/aos.v83.40686

A57 Janssens, L., Phlypo, I., Geddis-Regan, A., Petrovic, M. and Janssens, B. (2025) ‘Care home managers’ perspectives on domiciliary dental care: a qualitative study’, *BMC Geriatrics*, 25(1), p. 323. https://doi.org/10.1186/s12877...025...06005...5

A58 Langley, J., Wassall, R., Geddis-Regan, A., Watson, S., Verey, A., McKenna, G., Brocklehurst, P. and Tsakos, G. (2022) ‘Putting guidelines into practice: using co-design to develop a complex intervention based on NG48 to enable care staff to provide daily oral care to older people living in care homes’, *Gerodontology*, 40(1), p. 112. https://doi.org/10.1111/ger.12629

A59 Palmers, E., Janssens, L., Phlypo, I., Vanhaecht, K., De Almeida Mello, J., De Visschere, L., Declerck, D. and Duyck, J. (2022) ‘Perceptions on oral care needs, barriers, and practices among managers and staff in long-term care settings for older people in Flanders, Belgium: a cross-sectional survey’, *Innovation in Aging*, 6(5). https://doi.org/10.1093/geroni/igac046

A60 Rani, H., Azizan, A.A., Walad, N.I.A., Hassan, S.A.A., Dom, T.N.M., Yeoh, D.S.H., Tay, J.W.C., Rosli, M.S.A., Aun, N.S.M., Aziz, A.F.A., Thwin, K.M. and Tew, I.M. (2025) ‘Challenges and recommendations for oral healthcare of older adults in a long-term care facility’, *Healthcare*, 13(20), p. 2642. https://doi.org/10.3390/healthcare13202642

A61 Shigli, K., Nayak, S., Lagali-Jirge, V. and Karuveettil, V. (2025) ‘Oral healthcare experiences and needs of older adults in residential care facilities: a qualitative exploration of care managers’ perception in the Indian context’, *Gerodontology*. https://doi.org/10.1111/ger.70026

A62a Vandenbulcke, P.A.I., de Almeida Mello, J., Cornette, V., Brabants, M., Schoebrechts, E., De Lepeleire, J., Declercq, A., Declerck, D. and Duyck, J. (2024) ‘Nursing home residents’ perspectives on oral health: an in-depth interview study’, *International Journal of Nursing Studies Advances*, 6, p. 100198. https://doi.org/10.1016/j.ijnsa.2024.100198

A62b Vandenbulcke, P.A.I., de Almeida Mello, J., Piccart, F.M.P., De Lepeleire, J., Schoebrechts, E., Declercq, A., Declerck, D. and Duyck, J. (2025) ‘Implementation of the OHS-interRAI for regular oral health assessments by non-dental caregivers: focus group study using a SWOT analysis’, *BMC Public Health*, 25(1), p. 2552. https://doi.org/10.1186/s12889...025...22938...3

A63a Wong, F.M.F. and Leung, W.K. (2024) ‘Sustainability of an educational program on oral care/hygiene provision by healthcare providers to older residents in long-term care institutions: a follow-up study’, *Geriatrics*, 9(3), p. 84. https://doi.org/10.3390/geriatrics9030084

A63b Wong, F.M.F., Shie, H.W.H., Kao, E.N., Tsoi, H.M. and Leung, W.K. (2024) ‘Educational programme on knowledge, attitudes, and practice of oral care/hygiene provision by healthcare providers to older residents in long-term care institutions: a case-control study’, *Geriatrics*, 9(1), p. 16. https://doi.org/10.3390/geriatrics9010016

## Appendix B Supplementary materials

Image, application 1
