# Supplementary material for: Committing to oral care support for older people living in long-term residential care settings: A theoretically informed scoping review of enablers and barriers influencing care staff behaviours
Source: Int J Nurs Stud Adv. 2026 Aug 13;11:100658. doi: 10.1016/j.ijnsa.2026.100658 (PMC13578632; doi:10.1016/j.ijnsa.2026.100658)
Supplement: Supplementary file 1 [file mmc1.docx]

**Supplementary file 1: Search Strategies**

**Databases and sources searched**

**•** CINAHL (EBSCOhost)

**•** Core Collection (Web of Science): SCI-EXPANDED, SSCI, A&HCI, CPCI-S, CPCI-SSH and ESCI searched simultaneously

**•** Embase Classic+Embase (Ovid)

**•** Ovid MEDLINE(R) ALL

**•** APA PsycInfo (Ovid)

**Websites and other sources**

**•** British Society of Gerontology

**•** EThOS e-Thesis Online Service

**•** Google Scholar

**•** International Association for Dental Research Abstract Archives

**CINAHL (EBSCOhost)**

| ***Search*** | ***Search string*** |
| --- | --- |
| **S106** | S32 or S104 |
| **S105** | S32 or S104 |
| **S104** | S14 and S27 and S103 |
| **S103** | S33 OR S34 OR S35 OR S36 OR S37 OR S38 OR S39 OR S40 OR S41 OR S42 OR S43 OR S44 OR S45 OR S46 OR S47 OR S48 OR S49 OR S50 OR S51 OR S52 OR S53 OR S54 OR S55 OR S56 OR S57 OR S58 OR S59 OR S60 OR S61 OR S62 OR S63 OR S64 OR S65 OR S66 OR S67 OR S68 OR S69 OR S70 OR S71 OR S72 OR S73 OR S74 OR S75 OR S76 OR S77 OR S78 OR S79 OR S80 OR S81 OR S82 OR S83 OR S84 OR S85 OR S86 OR S87 OR S88 OR S89 OR S90 OR S91 OR S92 OR S93 OR S94 OR S95 OR S96 OR S97 OR S98 OR S99 OR S100 OR S101 OR S102 |
| **S102** | glaser* |
| **S101** | strauss* N2 corbin* |
| **S100** | Corbin* N2 strauss* |
| **S99** | Foucault* |
| **S98** | husserl* |
| **S97** | merleau N1 ponty* |
| **S96** | van N1 kaam* |
| **S95** | van N1 manen* |
| **S94** | Spiegelberg* |
| **S93** | Colaizzi* |
| **S92** | Heidegger* |
| **S91** | "narrative analysis" |
| **S90** | constant N1 comparison |
| **S89** | constant N1 comparative |
| **S88** | discurs* N3 analys?s |
| **S87** | discourse* N3 analys?s |
| **S86** | "content analysis" |
| **S85** | questionnaire* |
| **S84** | "observational method*" |
| **S83** | theme* or thematic |
| **S82** | "cluster sampl*" |
| **S81** | "life experience*" |
| **S80** | "lived experience*" |
| **S79** | "life world" or "life-world" or "conversation analys?s" or "personal experience*" or "theoretical saturation" |
| **S78** | account or accounts or unstructured or openended or "open ended" or text* or narrative* |
| **S77** | focus N1 group* |
| **S76** | purpos* N4 sampl* |
| **S75** | "theoretical sampl*" |
| **S74** | "biographical method" |
| **S73** | "human science" |
| **S72** | field N1 research |
| **S71** | field N1 stud* |
| **S70** | humanistic or existential or experiential or paradigm* |
| **S69** | "action research" or "cooperative inquir*" or "co operative inquir*" or "co-operative inquir*" |
| **S68** | "social construct*" or postmodern* or "post-structural*" or "post structural*" or poststructural* or "post modern*" or "post-modern*" or feminis* or interpret* |
| **S67** | "participant observ*" |
| **S66** | "data N1 saturat*" |
| **S65** | emic or etic or hermeneutic$ or heuristic$ or semiotic$ |
| **S64** | "women’s stor*" |
| **S63** | "life stor*" |
| **S62** | grounded N1 analys?s |
| **S61** | grounded N1 research |
| **S60** | grounded N1 studies |
| **S59** | grounded N1 study |
| **S58** | grounded N1 theor* |
| **S57** | phenomenol* |
| **S56** | ethnograph* |
| **S55** | Ethnonursing |
| **S54** | MH Cluster Sample+ |
| **S53** | MH Life Experiences+ |
| **S52** | MH Phenomenological Research |
| **S51** | MH Phenomenology |
| **S50** | MH theoretical sample |
| **S49** | MH Field Studies |
| **S48** | MH Observational Methods+ |
| **S47** | MH Purposive Sample |
| **S46** | MH Qualitative Validity+ |
| **S45** | MH Constant Comparative Method |
| **S44** | MH Ethnonursing Research |
| **S43** | MH Ethnological Research |
| **S42** | MH Ethnographic Research |
| **S41** | MH Content Analysis |
| **S40** | MH Discourse Analysis |
| **S39** | MH Focus Groups |
| **S38** | MH Questionnaires+ |
| **S37** | MH Research, Nursing |
| **S36** | MH Qualitative Studies |
| **S35** | MH Grounded theory |
| **S34** | (MH "Audiorecording") |
| **S33** | (MH "Interviews+") |
| **S32** | S14 and S27 and S31 |
| **S31** | S28 OR S29 OR S30 |
| **S30** | TI ( (barrier* or motivat* or challeng* or facilitat* or attitude* or perception* or view* or limit* or perspectiv* or opinion* or "adhere to" or adherence or compliance or comply*) ) OR AB ( (barrier* or motivat* or challeng* or facilitat* or attitude* or perception* or view* or limit* or perspectiv* or opinion* or "adhere to" or adherence or compliance or comply*) ) OR SU ( (barrier* or motivat* or challeng* or facilitat* or attitude* or perception* or view* or limit* or perspectiv* or opinion* or "adhere to" or adherence or compliance or comply*) ) |
| **S29** | (MH "Attitude") or (MH "Motivation+") |
| **S28** | (MH "Employee Attitudes") OR (MH "Attitude of Health Personnel") OR (MH "Nurse Attitudes") OR (MH "Caregiver Attitudes") OR (MH "Attitude to Health+") |
| **S27** | S21 OR S26 |
| **S26** | S24 AND S25 |
| **S25** | TI (prevent* or control* or reduc*) OR AB (prevent* or control* or reduc*) OR SU (prevent* or control* or reduc*) |
| **S24** | S22 OR S23 |
| **S23** | TX ( "oral disease*" or "oral neoplasm*" or "oral cancer*" or "mouth disease*" or "mouth neoplasm*" or "mouth cancer*" or "mouth ulcer#") OR TX ( "dental disease*" or "dental decay" or "dental plaque" or "oral plaque" ) OR TX ( (tooth or teeth) n2 (decay* or loss) ) OR TX ( "gum disease*" or DMF or caries or gingivitis or "periodontal disease*" or periodontitis or "dry mouth" or xerostomia ) |
| **S22** | TI ( ((oral or dent* or mouth or tongue or lip or lips or cheek or cheeks or teeth or tooth or gum or periodontal) n3 (abscess* or swelling*)) ) OR AB ( ((oral or dent* or mouth or tongue or lip or lips or cheek or cheeks or teeth or tooth or gum or periodontal) n3 (abscess* or swelling*)) ) OR SU ( ((oral or dent* or mouth or tongue or lip or lips or cheek or cheeks or teeth or tooth or gum or periodontal) n3 (abscess* or swelling*)) ) |
| **S21** | S15 OR S16 OR S17 OR S18 OR S19 OR S20 |
| **S20** | TX ( ((oral or dent* or mouth or tongue or lip or lips or cheek or cheeks or teeth or tooth or gum or periodontal) n3 (appointment* or consultation* or referral* or referred)) ) OR TX ( ((oral or dent* or mouth or tongue or lip or lips or cheek or cheeks or teeth or tooth or gum or periodontal) n3 (Promotion* or recommendation* or practice?)) ) |
| **S19** | TI ( denture# or (dental w1 (crown* or implant* or bridge* or inlay*)) ) OR TI ( ((tooth or teeth or plaque) n3 disclos*) ) AB ( denture# or (dental w1 (crown* or implant* or bridge* or inlay*)) ) OR AB ( ((tooth or teeth or plaque) n3 disclos*) ) OR SU ( denture# or (dental w1 (crown* or implant* or bridge* or inlay*)) ) OR SU ( ((tooth or teeth or plaque) n3 disclos*) ) |
| **S18** | TI ((oral or dental or mouth or tongue or lip or lips or cheek or cheeks or teeth or tooth or gum or periodontal) n1 (check* or assess* or exam* or screen*)) OR AB ((oral or dental or mouth or tongue or lip or lips or cheek or cheeks or teeth or tooth or gum or periodontal) n1 (check* or assess* or exam* or screen*)) OR SU ((oral or dental or mouth or tongue or lip or lips or cheek or cheeks or teeth or tooth or gum or periodontal) n1 (check* or assess* or exam* or screen*)) |
| **S17** | TX ( (mouthwash* or "mouth-wash*" or "mouth rins*" or mouthrins* or "oral rins*" or oralrins* ) OR TX ( toothpaste* or "tooth paste*" or dentifrice* or toothbrush* or "tooth brush*" or "fissure sealant*" or floss*) ) OR TX ( (fluorid* n2 (varnish* or topical or milk)) ) |
| **S16** | TI ( ((access* or availab*) n2 (dentist* or dental)) ) OR TI ( ((oral or dental or mouth or tongue or lip or lips or cheek or cheeks or teeth or tooth or gum or periodontal) w1 (care or hygiene or health )) ) OR AB ( ((access* or availab*) n2 (dentist* or dental)) ) OR AB ( ((oral or dental or mouth or tongue or lip or lips or cheek or cheeks or teeth or tooth or gum or periodontal) w1 (care or hygiene or health )) ) OR SU ( ((access* or availab*) n2 (dentist* or dental)) ) OR SU ( ((oral or dental or mouth or tongue or lip or lips or cheek or cheeks or teeth or tooth or gum or periodontal) w1 (care or hygiene or health )) ) |
| **S15** | ( (MH "Preventive Dentistry") OR (MH "Fluoridation") OR (MH "Mouthguards") OR (MH "Oral Hygiene+") OR (MH "Dental Prophylaxis+") ) OR ( (MH "Dental Health Education") OR (MH "Dental Care") OR (MH "Dental Care for Aged") OR (MH "Dental Care for Chronically Ill") OR (MH "Dental Care for Disabled") ) OR (MH "Stomatognathic Diseases+/PC") |
| **S14** | S10 OR S11 OR S12 OR S13 |
| **S13** | TI ( ((gerontol* or ageing or aging or elder* or geriatric* or senior# or "old age" or "late* life" or older) n2 (unit or units or center# or centre#)) ) OR AB ( ((gerontol* or ageing or aging or elder* or geriatric* or senior# or "old age" or "late* life" or older) n2 (unit or units or center# or centre#)) ) OR SU ( ((gerontol* or ageing or aging or elder* or geriatric* or senior# or "old age" or "late* life" or older) n2 (unit or units or center# or centre#)) ) |
| **S12** | TI ( ((gerontol* or ageing or aging or elder* or geriatric* or senior# or "old age" or "late* life" or older) n2 (home# or facilit* or institution* or ward#)). ) OR AB ( ((gerontol* or ageing or aging or elder* or geriatric* or senior# or "old age" or "late* life" or older) n2 (home# or facilit* or institution* or ward#)). ) OR SU ( ((gerontol* or ageing or aging or elder* or geriatric* or senior# or "old age" or "late* life" or older) n2 (home# or facilit* or institution* or ward#)). ) |
| **S11** | (MH "Aged, Hospitalized") OR TI "home# for the aged" OR AB "home# for the aged" |
| **S10** | S5 AND S9 |
| **S9** | S6 OR S7 OR S8 |
| **S8** | TI ( (Age? n3 (over or older) n2 (6# or 7# or 8# or 9#)) ) OR AB ( (Age? n3 (over or older) n2 (6# or 7# or 8# or 9#)) ) |
| **S7** | TX ( gerontol* or ageing or aging or elder* or geriatric* or senior# or "old age" or "late* life" ) OR TX ( older n1 (person* or people or adult* or patient* or m?n or wom?n or inpatient* or outpatient*) ) |
| **S6** | (MH "Aged+") or (MH "Geriatrics") |
| **S5** | S1 OR S2 OR S3 OR S4 |
| **S4** | TX ( (("long-term care" or "long-term stay" ) n3 (facility or facilities or setting or residen* or unit#)) ) OR TX ( (("intermediate care*" or "extended care*" ) n3 (facility or facilities or setting or residen* or home* or unit#)) ) OR TX ( ("skilled nursing*" n2 (facility or facilities or setting or residen* or care)) ) |
| **S3** | TX ( "rest home#" or hospice# or "assisted living facilit*" ) OR TX ( (residential n2 (care or setting#)) ) OR ( (rehab* n2 (facility or facilities or setting or residen* or care or home*)) ) OR TX ( (institutionali?ed n6 (adult# or patient# or individual#)) ) |
| **S2** | TI "care home#" OR AB "care home#" OR SU "care home#" OR TI ( ((nursing or residential) w2 (home# or facility or facilities)) ) OR AB ( ((nursing or residential) w2 (home# or facility or facilities)) ) OR SU ( ((nursing or residential) w2 (home# or facility or facilities)) ) |
| **S1** | (MH "Hospice Patients") OR (MH "Nursing Home Patients") OR (MH "Residential Facilities") OR (MH "Nursing Homes+") OR (MH "Hospices") OR (MH "Assisted Living") OR (MH "Long Term Care") |

**Core Collection (Web of Science)**

| **Search** | **Search string** |
| --- | --- |
| **24** | #22 OR #17 (publication-date limit applied; date values removed) |
| **23** | #22 OR #17 |
| **22** | #21 AND #15 AND #7 |
| **21** | #20 OR #19 OR #18 |
| **20** | "cluster sampl*" or ((lived or life) near/1 experience$) or "observational method*" or "content analysis" or (constant near/1 (comparative or comparison)) (Topic) or ((discourse* or discurs*) near/3 analys$s ) or narrative analys$s (Topic) |
| **19** | humanistic or existential or experiential or paradigm* or (field near/1 (study or studies or research)) (Topic) or (purpos* near/4 sampl*) or "focus group*" or account or accounts or unstructured or openended or "open ended" or text* or narrative* (Topic) or "life world" or life-world or "conversation analys$s" or "personal experience*" or "theoretical saturation" (Topic) |
| **18** | qualitative or theme* or thematic or questionnaire* or survey* or ethnograph* or phenomenol* or emic or etic or hermeneutic* or heuristic* or semiotic* (Topic) or (grounded near/1 (theor$ or study or studies or research or analys$s) ) (Topic) or (data near/1 saturat*) or "participant observ*" or social construct* or postmodern* or post-structural* or "post structural*" or poststructural* or "post modern*" or post-modern* or feminis* or interpret* (Topic) |
| **17** | #16 AND #15 AND #7 |
| **16** | barrier* or motivat* or challeng* or facilitat* or attitude* or perception* or view* or limit* or perspectiv* or opinion* (Topic) or "adhere to" or adherence or compliance or comply* (Topic) |
| **15** | #11 OR #14 |
| **14** | #13 AND #12 |
| **13** | prevent* or control* or reduc* (Topic) |
| **12** | "oral neoplasm*" or "oral cancer*" or "mouth neoplasm*" or "mouth cancer*" or "mouth ulcer$" or "dental decay" or "dental plaque" or "oral plaque" (Topic) or ((tooth or teeth) near/2 (decay* or loss)) or DMF or caries or gingivitis or periodontitis or "dry mouth" or xerostomia (Topic) or ((oral or dent* or mouth or tongue or lip or lips or cheek or cheeks or teeth or tooth or gum or periodontal) near/2 (disease* or abscess* or swelling*)) (Topic) |
| **11** | #8 OR #9 OR #10 |
| **10** | ((oral or dent* or mouth or tongue or lip or lips or cheek or cheeks or teeth or tooth or gum or periodontal) near/1 (check* or assess* or exam* or screen*)) (Topic) or ((oral or dent* or mouth or tongue or lip or lips or cheek or cheeks or teeth or tooth or gum or periodontal) near/3 (appointment* or consultation* or referral* or referred or Promotion* or recommendation* or practice$)) (Topic) |
| **9** | (fluorid* near/2 (varnish* or topical or milk)) (Topic) or (dental near/1 (crown* or implant* or bridge* or inlay*)) (Topic) or (tooth or teeth or plaque) near/3 disclos* (Topic) or denture$ (Topic) |
| **8** | ((access* or availab*) near/2 (dentist* or dental)) (Topic) or ((oral or dent* or mouth or tongue or lip or lips or cheek or cheeks or teeth or tooth or gum or periodontal) near/1 (care or hygiene or health)) (Topic) or mouthwash* or mouth-wash* or mouth-rins* or mouthrins* or "oral rins*" or oralrins* or toothpaste* or "tooth paste*" or dentifrice* or toothbrush* or "tooth brush*" or "fissure sealant*" (Topic) |
| **7** | #6 OR #5 |
| **6** | "home$ for the aged" (Topic) or ((gerontol* or ageing or aging or elder* or geriatric* or senior$ or "old age" or "late* life" or older) near/2 (home$ or facilit* or institution* or ward or wards)) (Topic) or ((gerontol* or ageing or aging or elder* or geriatric* or senior$ or "old age" or "late* life" or older) near/2 (centre$ or center$ or unit$)) (Topic) |
| **5** | #4 AND #3 |
| **4** | older near/1 (person* or people or adult* or patient* or men or man or women or woman or inpatient* or outpatient*) (Topic) or gerontol* or ageing or aging or elder* or geriatric* or senior$ or "old age" or "late* life" (Topic) or (Age$ near/3 (over or older) near/2 (65 or 70 or 75 or 80 or 90)) (Topic) |
| **3** | #2 OR #1 |
| **2** | (("long-term care" or "long-term stay") near/3 (facility or facilities or setting or residen* or unit$)) (Topic) or (("intermediate care*" or "extended care*" or "skilled nursing") near/3 (facility or facilities or setting or residen* or home* or unit$)) (Title) |
| **1** | (nursing or residential) near/2 (home$ or facility or facilities) (Topic) or "care home$" or hospice$ (Topic) or residential near/2 (care or setting$) (Topic) |

**Embase Classic+Embase (Ovid)**

| ***Search*** | ***Search string*** |
| --- | --- |
| 1 | nursing home/ |
| 2 | residential home/ |
| 3 | hospice/ |
| 4 | hospice nursing/ |
| 5 | care home?.tw,kf. |
| 6 | rest home?.tw,kf. |
| 7 | hospice?.tw,kf. |
| 8 | *long term care/ |
| 9 | assisted living facility/ |
| 10 | assisted living facilit*.tw,kf. |
| 11 | (residential adj2 (care or setting?)).tw,kf. |
| 12 | (rehab* adj2 (facility or facilities or setting or residen* or care or home*)).tw,kf. |
| 13 | (institutionali#ed adj6 (adult? or patient? or individual?)).tw,kf. |
| 14 | ("long-term care" adj3 (facility or facilities or setting or residen* or unit?)).tw,kf. |
| 15 | ("long-term stay" adj3 (facility or facilities or setting or residen* or unit?)).tw,kf. |
| 16 | (intermediate care* adj3 (facility or facilities or setting or residen* or home* or unit?)).tw,kf. |
| 17 | (extended care* adj3 (facility or facilities or setting or residen* or home* or unit?)).tw,kf. |
| 18 | (skilled nursing* adj2 (facility or facilities or setting or residen* or care)).tw,kf. |
| 19 | or/1-18 [Care home or long-term similar settings] |
| 20 | (older adj (person* or people or adult* or patient* or m#n or wom#n or inpatient* or outpatient*)).tw,kf. |
| 21 | (gerontol* or ageing or aging or elder* or geriatric* or senior? or old age or late* life).tw,kf. |
| 22 | (Age? adj3 (over or older) adj2 (6# or 7# or 8# or 9#)).tw. |
| 23 | (">=6# years old" or ">6# years old").tw. |
| 24 | (">=7# years old" or ">7# years old").tw. |
| 25 | (">=8# years old" or ">8# years old").tw. |
| 26 | (">=9# years old" or ">9# years old").tw. |
| 27 | (Age? adj3 (over or older) adj2 (6# or 7# or 8# or 9#)).tw. |
| 28 | aged/ or frail elderly/ or institutionalized elderly/ or very elderly/ |
| 29 | exp geriatrics/ |
| 30 | or/20-29 [Over 65 yrs old] |
| 31 | 19 and 30 [Care home and older people search 1 65yrs] |
| 32 | home for the aged/ |
| 33 | ((gerontol* or ageing or aging or elder* or geriatric* or senior? or old age or late* life or older) adj2 home?).tw,kf. |
| 34 | ((gerontol* or ageing or aging or elder* or geriatric* or senior? or old age or late* life or older) adj2 facilit*).tw,kf. |
| 35 | ((gerontol* or ageing or aging or elder* or geriatric* or senior? or old age or late* life or older) adj2 institution*).tw,kf. |
| 36 | ((gerontol* or ageing or aging or elder* or geriatric* or senior? or old age or late* life or older) adj2 (ward or wards or unit or units)).tw,kf. |
| 37 | ((gerontol* or ageing or aging or elder* or geriatric* or senior? or old age or late* life or older) adj2 (centre? or center?)).tw,kf. |
| 38 | "home? for the aged".tw,kf. |
| 39 | or/32-38 [Care home older people search 2] |
| 40 | 31 or 39 [Care home over 60s combined search] |
| 41 | preventive dentistry/ |
| 42 | mouth hygiene/ |
| 43 | dental prevention/ or caries prevention/ or tooth brushing/ |
| 44 | mouthwash/ |
| 45 | dental health education/ |
| 46 | ((access* or availab*) adj2 (dentist* or dental)).tw,kf. |
| 47 | ((oral or dent* or mouth or tongue or lip or lips or cheek or cheeks or teeth or tooth or gum or periodontal) adj care).tw,kf. |
| 48 | ((oral or dent* or mouth or tongue or lip or lips or cheek or cheeks or teeth or tooth or gum or periodontal) adj hygiene).tw,kf. |
| 49 | ((oral or dent* or mouth or tongue or lip or lips or cheek or cheeks or teeth or tooth or gum or periodontal) adj health).tw,kf. |
| 50 | (mouthwash* or mouth-wash* or mouth-rins* or mouthrins* or oral rins* or oralrins*).tw,kf. |
| 51 | (toothpaste* or tooth paste* or dentifrice* or toothbrush* or tooth brush* or fissure sealant* or floss*).tw,kf. |
| 52 | (fluorid* adj2 (varnish* or topical or milk)).tw,kf. |
| 53 | dental floss/ |
| 54 | fluoride varnish/ |
| 55 | fissure sealant/ |
| 56 | exp mouth disease/pc [Prevention] |
| 57 | (dental adj (crown* or implant* or bridge* or inlay*)).tw,kf. |
| 58 | denture?.tw,kf. |
| 59 | ((oral or dent* or mouth or tongue or lip or lips or cheek or cheeks or teeth or tooth or gum or periodontal) adj1 check*).tw,kf. |
| 60 | ((oral or dent* or mouth or tongue or lip or lips or cheek or cheeks or teeth or tooth or gum or periodontal) adj1 assess*).tw,kf. |
| 61 | ((oral or dent* or mouth or tongue or lip or lips or cheek or cheeks or teeth or tooth or gum or periodontal) adj1 exam*).tw,kf. |
| 62 | ((oral or dent* or mouth or tongue or lip or lips or cheek or cheeks or teeth or tooth or gum or periodontal) adj1 screen*).tw,kf. |
| 63 | ((tooth or teeth or plaque) adj3 disclos*).tw,kf. |
| 64 | ((oral or dent* or mouth or tongue or lip or lips or cheek or cheeks or teeth or tooth or gum or periodontal) adj3 (Promotion* or recommendation* or Practice?)).tw,kf. |
| 65 | ((oral or dent* or mouth or tongue or lip or lips or cheek or cheeks or teeth or tooth or gum or periodontal) adj3 (appointment* or consultation* or referral* or referred)).tw,kf. |
| 66 | or/41-65 [Prevention and Control of Dental Problems] |
| 67 | (oral disease* or oral neoplasm* or oral cancer* or mouth disease* or mouth neoplasm* or mouth cancer* or mouth ulcer?).tw,kf. |
| 68 | (dental disease* or dental decay or dental plaque or oral plaque).tw,kf. |
| 69 | ((tooth or teeth) adj2 (decay* or loss)).tw,kf. |
| 70 | (gum disease* or DMF or caries or gingivitis or periodontal disease* or periodontitis or dry mouth or xerostomia).tw,kf. |
| 71 | ((oral or dent* or mouth or tongue or lip or lips or cheek or cheeks or teeth or tooth or gum or periodontal) adj3 (abscess* or swelling*)).tw,kf. |
| 72 | or/67-71 [Dental Problems] |
| 73 | (prevent* or control* or reduc*).tw. |
| 74 | pc.fs. |
| 75 | 73 or 74 |
| 76 | 72 and 75 [Prevention of Dental Problems] |
| 77 | 66 or 76 [Oral Care or Prevention of Dental Problems] |
| 78 | 40 and 77 [Care home over 60s Oral Care] |
| 79 | exp qualitative research/ |
| 80 | exp interview/ |
| 81 | interview*.tw,kw. |
| 82 | qualitative.tw,kw. |
| 83 | nursing methodology research/ |
| 84 | questionnaire*.mp. |
| 85 | survey*.mp. |
| 86 | ethnolog*.mp. |
| 87 | ethnograph*.mp. |
| 88 | ethnonursing.af. |
| 89 | phenomenol*.af. |
| 90 | (grounded adj (theor$ or study or studies or research or analys?s)).af. |
| 91 | (life stor$ or women* stor$).mp. |
| 92 | (emic or etic or hermeneutic* or heuristic* or semiotic*).af. or (data adj1 saturat*).tw. or participant observ*.tw. |
| 93 | (social construct$ or postmodern$ or post-structural$ or poststructural$ or post-modern$ or feminis$ or interpret$).mp. |
| 94 | (action research or cooperative inquir* or co-operative inquir*).mp. |
| 95 | (humanistic or existential or experiential or paradigm$).mp. |
| 96 | (field adj (study or studies or research)).tw. |
| 97 | human science.tw. |
| 98 | biographical method.tw. |
| 99 | theoretical sampl$.af. |
| 100 | ((purpos* adj4 sampl*) or (focus adj group*)).af. |
| 101 | (account or accounts or unstructured or openended or open ended or text$ or narrative$).mp. |
| 102 | (life world or life-world or conversation analys?s or personal experience* or theoretical saturation).mp. |
| 103 | ((lived or life) adj experience$).mp. |
| 104 | cluster sampl*.mp. |
| 105 | observational method*.af. |
| 106 | content analysis.af. |
| 107 | (constant adj (comparative or comparison)).af. |
| 108 | ((discourse* or discurs*) adj3 analys?s).tw. |
| 109 | narrative analys?s.af. |
| 110 | heidegger$.tw. |
| 111 | (van adj kaam$).tw. |
| 112 | colaizzi$.tw. |
| 113 | spiegelberg$.tw. |
| 114 | (van adj manen$).tw. |
| 115 | (merleau adj ponty$).tw. |
| 116 | husserl$.tw. |
| 117 | foucault$.tw. |
| 118 | (corbin$ adj2 strauss$).tw. |
| 119 | glaser$.tw. |
| 120 | or/79-119 [Adapted De Jean et al. 2016 Hybrid Ovid Medline Qualitative filter] |
| 121 | 78 and 120 [Care home over 60s Oral Care De Jean Qual filter] |
| 122 | limit 78 to "qualitative (maximizes sensitivity)" |
| 123 | exp motivation/ |
| 124 | (barrier* or motivat* or challeng* or facilitat* or attitude* or perception* or view* or limit* or perspectiv* or opinion*).tw,kf. |
| 125 | ("adhere to" or adherence or compliance or comply*).tw,kf. |
| 126 | exp attitude/ |
| 127 | or/123-126 [Barriers Facilitators Attitudes] |
| 128 | 78 and 127 [Care Home oral care AND Barriers Facilitators Attitudes] |
| 129 | 121 or 122 or 128 [Care home oral care qualitative studies or barriers studies] |
| 130 | limit 129 to yr=[publication-date range removed] |

**Ovid MEDLINE(R) ALL**

| ***Search*** | ***Search string*** |
| --- | --- |
| 1 | exp Nursing Homes/ |
| 2 | Residential Facilities/ |
| 3 | Hospices/ |
| 4 | care home?.tw,kf. |
| 5 | ((nursing or residential) adj2 (home? or facility or facilities)).tw,kf. |
| 6 | rest home?.tw,kf. |
| 7 | hospice?.tw,kf. |
| 8 | Long-Term Care/ |
| 9 | Assisted Living Facilities/ |
| 10 | assisted living facilit*.tw,kf. |
| 11 | (residential adj2 (care or setting?)).tw,kf. |
| 12 | (rehab* adj2 (facility or facilities or setting or residen* or care or home*)).tw,kf. |
| 13 | (institutionali#ed adj6 (adult? or patient? or individual?)).tw,kf. |
| 14 | ("long-term care" adj3 (facility or facilities or setting or residen* or unit?)).tw,kf. |
| 15 | ("long-term stay" adj3 (facility or facilities or setting or residen* or unit?)).tw,kf. |
| 16 | (intermediate care* adj3 (facility or facilities or setting or residen* or home* or unit?)).tw,kf. |
| 17 | (extended care* adj3 (facility or facilities or setting or residen* or home* or unit?)).tw,kf. |
| 18 | (skilled nursing* adj2 (facility or facilities or setting or residen* or care)).tw,kf. |
| 19 | or/1-18 [Care home or long-term similar settings] |
| 20 | exp aged/ |
| 21 | exp geriatrics/ |
| 22 | (older adj (person* or people or adult* or patient* or m#n or wom#n or inpatient* or outpatient*)).tw,kf. |
| 23 | (gerontol* or ageing or aging or elder* or geriatric* or senior? or old age or late* life).tw,kf. |
| 24 | (Age? adj3 (over or older) adj2 (6# or 7# or 8# or 9#)).tw. |
| 25 | (">=6# years old" or ">6# years old").tw. |
| 26 | (">=7# years old" or ">7# years old").tw. |
| 27 | (">=8# years old" or ">8# years old").tw. |
| 28 | (">=9# years old" or ">9# years old").tw. |
| 29 | (Age? adj3 (over or older) adj2 (6# or 7# or 8# or 9#)).tw. |
| 30 | or/20-29 [Older People over 60yrs] |
| 31 | 30 and 19 [Care home and older people search 1 60yrs] |
| 32 | Homes for the Aged/ |
| 33 | "home? for the aged".tw,kf. |
| 34 | ((gerontol* or ageing or aging or elder* or geriatric* or senior? or old age or late* life or older) adj2 home?).tw,kf. |
| 35 | ((gerontol* or ageing or aging or elder* or geriatric* or senior? or old age or late* life or older) adj2 facilit*).tw,kf. |
| 36 | ((gerontol* or ageing or aging or elder* or geriatric* or senior? or old age or late* life or older) adj2 institution*).tw,kf. |
| 37 | ((gerontol* or ageing or aging or elder* or geriatric* or senior? or old age or late* life or older) adj2 (ward or wards or unit or units)).tw,kf. |
| 38 | ((gerontol* or ageing or aging or elder* or geriatric* or senior? or old age or late* life or older) adj2 (centre? or center?)).tw,kf. |
| 39 | or/32-38 [Care home older people search 2] |
| 40 | 31 or 39 [Care home over 60s combined search] |
| 41 | Preventive dentistry/ |
| 42 | Oral Hygiene/ |
| 43 | Dental Care/ |
| 44 | Toothbrushing/ |
| 45 | Mouthwashes/ |
| 46 | Health Education, Dental/ |
| 47 | Oral health/ |
| 48 | Dental Care for Chronically Ill/ |
| 49 | Dental Care for Aged/ |
| 50 | Dental Care for Disabled/ |
| 51 | Geriatric Dentistry/ |
| 52 | ((access* or availab*) adj2 (dentist* or dental)).tw,kf. |
| 53 | ((oral or dent* or mouth or tongue or lip or lips or cheek or cheeks or teeth or tooth or gum or periodontal) adj care).tw,kf. |
| 54 | ((oral or dent* or mouth or tongue or lip or lips or cheek or cheeks or teeth or tooth or gum or periodontal) adj hygiene).tw,kf. |
| 55 | ((oral or dent* or mouth or tongue or lip or lips or cheek or cheeks or teeth or tooth or gum or periodontal) adj health).tw,kf. |
| 56 | (mouthwash* or mouth-wash* or mouth-rins* or mouthrins* or oral rins* or oralrins*).tw,kf. |
| 57 | (toothpaste* or tooth paste* or dentifrice* or toothbrush* or tooth brush* or fissure sealant* or floss*).tw,kf. |
| 58 | exp Dentifrices/ |
| 59 | (fluorid* adj2 (varnish* or topical or milk)).tw,kf. |
| 60 | Fluorides, Topical/ |
| 61 | exp Stomatognathic Diseases/pc [Prevention & Control] |
| 62 | (dental adj (crown* or implant* or bridge* or inlay*)).tw,kf. |
| 63 | denture?.tw,kf. |
| 64 | ((oral or dent* or mouth or tongue or lip or lips or cheek or cheeks or teeth or tooth or gum or periodontal) adj1 check*).tw,kf. |
| 65 | ((oral or dent* or mouth or tongue or lip or lips or cheek or cheeks or teeth or tooth or gum or periodontal) adj1 assess*).tw,kf. |
| 66 | ((oral or dent* or mouth or tongue or lip or lips or cheek or cheeks or teeth or tooth or gum or periodontal) adj1 exam*).tw,kf. |
| 67 | ((oral or dent* or mouth or tongue or lip or lips or cheek or cheeks or teeth or tooth or gum or periodontal) adj1 screen*).tw,kf. |
| 68 | ((tooth or teeth or plaque) adj3 disclos*).tw,kf. |
| 69 | ((oral or dent* or mouth or tongue or lip or lips or cheek or cheeks or teeth or tooth or gum or periodontal) adj3 (appointment* or consultation* or referral* or referred)).tw,kf. |
| 70 | ((oral or dent* or mouth or tongue or lip or lips or cheek or cheeks or teeth or tooth or gum or periodontal) adj3 (Promotion* or recommendation* or practice?)).tw,kf. |
| 71 | or/41-70 [Prevention and Control of Dental Problems] |
| 72 | (oral disease* or oral neoplasm* or oral cancer* or mouth disease* or mouth neoplasm* or mouth cancer* or "mouth ulcer?").tw,kf. |
| 73 | (dental disease* or dental decay or dental plaque or oral plaque).tw,kf. |
| 74 | ((tooth or teeth) adj2 (decay* or loss)).tw,kf. |
| 75 | (gum disease* or DMF or caries or gingivitis or periodontal disease* or periodontitis or dry mouth or xerostomia).tw,kf. |
| 76 | ((oral or dent* or mouth or tongue or lip or lips or cheek or cheeks or teeth or tooth or gum or periodontal) adj3 (abscess* or swelling*)).tw,kf. |
| 77 | or/72-76 [Dental Problems] |
| 78 | (prevent* or control* or reduc*).tw. |
| 79 | pc.fs. |
| 80 | 78 or 79 |
| 81 | 77 and 80 [Prevention of Dental Problems] |
| 82 | 71 or 81 [Oral Care or Prevention of Dental Problems] |
| 83 | 40 and 82 [Care home over 60s Oral Care] |
| 84 | exp qualitative research/ |
| 85 | "Surveys and Questionnaires"/ |
| 86 | interview/ |
| 87 | (theme* or thematic).mp. |
| 88 | qualitative.af. |
| 89 | Nursing Methodology Research/ |
| 90 | questionnaire*.mp. |
| 91 | survey*.mp. |
| 92 | ethnological research.mp. |
| 93 | ethnograph*.mp. |
| 94 | ethnonursing.af. |
| 95 | phenomenol*.af. |
| 96 | (grounded adj (theor$ or study or studies or research or analys?s)).af. |
| 97 | (life stor or women* stor*).mp. |
| 98 | (emic or etic or hermeneutic* or heuristic* or semiotic*).af. or (data adj1 saturat*).tw. or participant observ*.tw. |
| 99 | (social construct* or (postmodern* or post-structural*) or (post structural* or poststructural*) or post modern* or post-modern* or feminis* or interpret*).mp. |
| 100 | (action research or cooperative inquir* or co operative inquir* or co-operative inquir*).mp. |
| 101 | (humanistic or existential or experiential or paradigm*).mp. |
| 102 | (field adj (study or studies or research)).tw. |
| 103 | human science.tw. |
| 104 | biographical method.tw. |
| 105 | theoretical sampl*.af. |
| 106 | ((purpos* adj4 sampl*) or (focus adj group*)).af. |
| 107 | (account or accounts or unstructured or openended or open ended or text* or narrative*).mp. |
| 108 | (life world or life-world or conversation analys?s or personal experience* or theoretical saturation).mp. |
| 109 | cluster sampl*.mp. |
| 110 | ((lived or life) adj experience$).mp. |
| 111 | observational method*.af. |
| 112 | content analysis.af. |
| 113 | (constant adj (comparative or comparison)).af. |
| 114 | ((discourse* or discurs*) adj3 analys?s).tw. |
| 115 | narrative analys?s.af. |
| 116 | heidegger*.tw. |
| 117 | colaizzi*.tw. |
| 118 | spiegelberg*.tw. |
| 119 | (van adj manen*).tw. |
| 120 | (van adj kaam*).tw. |
| 121 | (merleau adj ponty*).tw. |
| 122 | husserl*.tw. |
| 123 | foucault*.tw. |
| 124 | (corbin$ adj2 strauss*).tw. |
| 125 | glaser*.tw. |
| 126 | or/84-125 [De Jean Qualitative Studies Filter] |
| 127 | 83 and 126 [Care home over 60s Oral Care De Jean Qual filter] |
| 128 | limit 83 to "qualitative (maximizes sensitivity)" [Care home over 60's Medline Sensitive Qual filter] |
| 129 | 127 or 128 [Care home oral care with either Qual filter] |
| 130 | exp Motivation/ |
| 131 | exp "Attitude of Health Personnel"/ |
| 132 | "treatment adherence and compliance"/ or exp "patient acceptance of health care"/ or exp patient satisfaction/ or exp treatment refusal/ |
| 133 | Attitude/ |
| 134 | (barrier* or motivat* or challeng* or facilitat* or attitude* or perception* or view* or limit* or perspectiv* or opinion*).tw,kf. |
| 135 | ("adhere to" or adherence or compliance or comply*).tw,kf. |
| 136 | or/130-135 [Barriers Facilitators Attitudes] |
| 137 | 83 and 136 [Care Home oral care AND Barriers Facilitators Attitudes] |
| 138 | 137 or 129 [Care home oral care qualitative studies or barriers studies] |
| 139 | limit 138 to yr=[publication-date range removed] [Care home oral care qualitative studies or barriers studies; date values removed] |

**APA PsycInfo (Ovid)**

| ***Search*** | ***Search string*** |
| --- | --- |
| 1 | residential care institutions/ or exp nursing homes/ |
| 2 | hospice/ |
| 3 | care home?.tw,id. |
| 4 | ((nursing or residential) adj2 (home? or facility or facilities)).tw,id. |
| 5 | rest home?.tw,id. |
| 6 | hospice?.tw,id. |
| 7 | long term care/ |
| 8 | assisted living/ |
| 9 | assisted living facilit*.tw,id. |
| 10 | (residential adj2 (care or setting?)).tw,id. |
| 11 | (rehab* adj2 (facility or facilities or setting or residen* or care or home*)).tw,id. |
| 12 | (institutionali#ed adj6 (adult? or patient? or individual?)).tw,id. |
| 13 | ("long-term care" adj3 (facility or facilities or setting or residen* or unit?)).tw,id. |
| 14 | ("long-term stay" adj3 (facility or facilities or setting or residen* or unit?)).tw,id. |
| 15 | (intermediate care* adj3 (facility or facilities or setting or residen* or home* or unit?)).tw,id. |
| 16 | (extended care* adj3 (facility or facilities or setting or residen* or home* or unit?)).tw,id. |
| 17 | (skilled nursing* adj2 (facility or facilities or setting or residen* or care)).tw,id. |
| 18 | or/1-17 [Care home or long-term similar settings] |
| 19 | aged 65 yrs older.ag. |
| 20 | exp geriatrics/ |
| 21 | (older adj (person* or people or adult* or patient* or m#n or wom#n or inpatient* or outpatient*)).tw,id. |
| 22 | (Age? adj3 (over or older) adj2 (6# or 7# or 8# or 9#)).tw. |
| 23 | (gerontol* or ageing or aging or elder* or geriatric* or senior? or old age or late* life).tw,id. |
| 24 | (">=6# years old" or ">6# years old").tw. |
| 25 | (">=7# years old" or ">7# years old").tw. |
| 26 | (">=8# years old" or ">8# years old").tw. |
| 27 | (">=9# years old" or ">9# years old").tw. |
| 28 | (Age? adj3 (over or older) adj2 (6# or 7# or 8# or 9#)).tw. |
| 29 | or/19-28 [Older People over 60yrs] |
| 30 | 18 and 29 [Care home and older people search 1 60yrs] |
| 31 | ((gerontol* or ageing or aging or elder* or geriatric* or senior? or old age or late* life or older) adj2 home?).tw,id. |
| 32 | ((gerontol* or ageing or aging or elder* or geriatric* or senior? or old age or late* life or older) adj2 facilit*).tw,id. |
| 33 | ((gerontol* or ageing or aging or elder* or geriatric* or senior? or old age or late* life or older) adj2 institution*).tw,id. |
| 34 | ((gerontol* or ageing or aging or elder* or geriatric* or senior? or old age or late* life or older) adj2 (ward or wards or unit or units)).tw,id. |
| 35 | ((gerontol* or ageing or aging or elder* or geriatric* or senior? or old age or late* life or older) adj2 (centre? or center?)).tw,id. |
| 36 | "home? for the aged".tw,id. |
| 37 | or/31-36 [Care home older people search 2] |
| 38 | 30 or 37 [Care home over 60s combined search] |
| 39 | dentistry/ |
| 40 | oral health/ or dental health/ |
| 41 | ((access* or availab*) adj2 (dentist* or dental)).tw,id. |
| 42 | ((oral or dent* or mouth or tongue or lip or lips or cheek or cheeks or teeth or tooth or gum or periodontal) adj care).tw,id. |
| 43 | ((oral or dent* or mouth or tongue or lip or lips or cheek or cheeks or teeth or tooth or gum or periodontal) adj hygiene).tw,id. |
| 44 | ((oral or dent* or mouth or tongue or lip or lips or cheek or cheeks or teeth or tooth or gum or periodontal) adj health).tw,id. |
| 45 | (mouthwash* or mouth-wash* or mouth-rins* or mouthrins* or oral rins* or oralrins*).tw,id. |
| 46 | (fluorid* adj2 (varnish* or topical or milk)).tw,id. |
| 47 | (dental adj (crown* or implant* or bridge* or inlay*)).tw,id. |
| 48 | denture?.tw,id. |
| 49 | ((oral or dent* or mouth or tongue or lip or lips or cheek or cheeks or teeth or tooth or gum or periodontal) adj1 check*).tw,id. |
| 50 | ((oral or dent* or mouth or tongue or lip or lips or cheek or cheeks or teeth or tooth or gum or periodontal) adj1 assess*).tw,id. |
| 51 | ((oral or dent* or mouth or tongue or lip or lips or cheek or cheeks or teeth or tooth or gum or periodontal) adj1 exam*).tw,id. |
| 52 | ((oral or dent* or mouth or tongue or lip or lips or cheek or cheeks or teeth or tooth or gum or periodontal) adj1 screen*).tw,id. |
| 53 | ((tooth or teeth or plaque) adj3 disclos*).tw,id. |
| 54 | ((oral or dent* or mouth or tongue or lip or lips or cheek or cheeks or teeth or tooth or gum or periodontal) adj3 (Promotion* or recommendation* or Practice?)).tw,id. |
| 55 | ((oral or dent* or mouth or tongue or lip or lips or cheek or cheeks or teeth or tooth or gum or periodontal) adj3 (appointment* or consultation* or referral* or referred)).tw,id. |
| 56 | or/39-55 [Prevention and Control of Dental Problems] |
| 57 | (oral disease* or oral neoplasm* or oral cancer* or mouth disease* or mouth neoplasm* or mouth cancer* or mouth ulcer*).tw,id. |
| 58 | (dental disease* or dental decay or dental plaque or oral plaque).tw,id. |
| 59 | ((tooth or teeth) adj2 (decay* or loss)).tw,id. |
| 60 | (gum disease* or DMF or caries or gingivitis or periodontal disease* or periodontitis or dry mouth or xerostomia).tw,id. |
| 61 | ((oral or dent* or mouth or tongue or lip or lips or cheek or cheeks or teeth or tooth or gum or periodontal) adj3 (abscess* or swelling*)).tw,id. |
| 62 | or/57-61 [Dental Problems] |
| 63 | (prevent* or control* or reduc*).tw. |
| 64 | 62 and 63 [Prevention of Dental Problems] |
| 65 | 56 or 64 [Oral Care or Prevention of Dental Problems] |
| 66 | 38 and 65 |
| 67 | interview.af. |
| 68 | (theme* or thematic).mp. |
| 69 | qualitative.af. |
| 70 | Methodol* Research.tw. |
| 71 | questionnaire*.mp. |
| 72 | survey*.mp. |
| 73 | ethnological research.mp. |
| 74 | ethnograph*.mp. |
| 75 | ethnonursing.af. |
| 76 | phenomenol*.af. |
| 77 | (grounded adj (theor$ or study or studies or research or analys?s)).af. |
| 78 | (life stor or women* stor*).mp. |
| 79 | (emic or etic or hermeneutic* or heuristic* or semiotic*).af. or (data adj1 saturat*).tw. or participant observ*.tw. |
| 80 | (social construct* or postmodern* or post-structural* or poststructural* or post-modern* or feminis* or interpret*).mp. |
| 81 | (action research or cooperative inquir* or co-operative inquir*).mp. |
| 82 | (humanistic or existential or experiential or paradigm*).mp. |
| 83 | (field adj (study or studies or research)).tw. |
| 84 | human science.tw. |
| 85 | biographical method.tw. |
| 86 | theoretical sampl*.af. |
| 87 | ((purpos* adj4 sampl*) or (focus adj group*)).af. |
| 88 | (account or accounts or unstructured or openended or open ended or text* or narrative*).mp. |
| 89 | (life world or life-world or conversation analys?s or personal experience* or theoretical saturation).mp. |
| 90 | cluster sampl*.mp. |
| 91 | ((lived or life) adj experience$).mp. |
| 92 | observational method*.af. |
| 93 | content analysis.af. |
| 94 | (constant adj (comparative or comparison)).af. |
| 95 | ((discourse* or discurs*) adj3 analys?s).tw. |
| 96 | narrative analys?s.af. |
| 97 | heidegger*.tw. |
| 98 | colaizzi*.tw. |
| 99 | spiegelberg*.tw. |
| 100 | (van adj manen*).tw. |
| 101 | (van adj kaam*).tw. |
| 102 | (merleau adj ponty*).tw. |
| 103 | husserl*.tw. |
| 104 | foucault*.tw. |
| 105 | (corbin$ adj2 strauss*).tw. |
| 106 | glaser*.tw. |
| 107 | or/67-106 [De Jean Qualitative Filter] |
| 108 | 66 and 107 |
| 109 | limit 66 to "qualitative (maximizes sensitivity)" |
| 110 | exp motivation/ |
| 111 | exp attitudes/ |
| 112 | (barrier* or motivat* or challeng* or facilitat* or attitude* or perception* or view* or limit* or perspectiv* or opinion*).tw,in. |
| 113 | ("adhere to" or adherence or compliance or comply*).tw,in. |
| 114 | or/110-113 [Barriers Facilitators Attitudes] |
| 115 | 66 and 114 |
| 116 | 108 or 109 or 115 |
| 117 | limit 116 to yr=[publication-date range removed] |

**British Society of Gerontology**

https://www.britishgerontology.org/

Each phrase or term was searched separately in the website search box.

Search string(s)

“oral care”

“oral hygiene”

“mouth care”

dental

**EThOS e-Thesis Online Service**

https://ethos.bl.uk/Home.do

Each search was conducted separately.

Search string(s)

“care home” AND “oral”

“care home” AND “mouth care”

“care home” AND dental

“nursing home” AND oral

“nursing home” AND “mouth care”

“nursing home” AND dental

“residential care” AND oral

“residential care” AND “mouth care”

“residential care” AND dental

“long term care” AND oral

“long term care” AND “mouth care”

“long term care” AND dental

**Google Scholar**

https://scholar.google.co.uk/

Search run in Harzing’s Publish or Perish. A publication-date limit was applied; date values are removed.

Search string(s)

"care homes"|"nursing homes"|"residential care" AND "oral health"|"oral hygiene"|"oral care"|"mouth care"|dental AND barriers|facilitators|motivate|challenge|facilitate|attitude|qualitative|survey|interview|questionnaire|adherence|compliance

**International Association for Dental Research Abstract Archives**

https://iadr.abstractarchives.com/search

Using the Advanced Search Form, each phrase was searched separately in “Abstract Title, Body, Authors & Categories”. A publication-date limit was applied; date values are removed.

Search string(s)

“care homes”

“nursing homes”

“residential care”

“long term care”
